# Noncovalent Conjugation of OVA323 to ELP Micelles Increases Immune Response

**DOI:** 10.1021/acs.biomac.3c01091

**Published:** 2024-01-03

**Authors:** Jolinde van Strien, Max Makurat, Ye Zeng, René Olsthoorn, Gregory F. Schneider, Bram Slütter, J. Andrew MacKay, Wim Jiskoot, Alexander Kros

**Affiliations:** † Department of Supramolecular and Biomaterials Chemistry, Leiden Institute of Chemistry, Leiden University, P.O. Box 9502, 2300 RA Leiden, The Netherlands; ‡ Department of BioTherapeutics, LACDR, Leiden University, P.O. Box 9502, 2300 RA Leiden, The Netherlands; § Department of Pharmacology and Pharmaceutical Sciences, Alfred E. Mann School of Pharmacy and Pharmaceutical Sciences, University of Southern California, 1985 Zonal Avenue, Los Angeles, California 90089-9121, United States

## Abstract

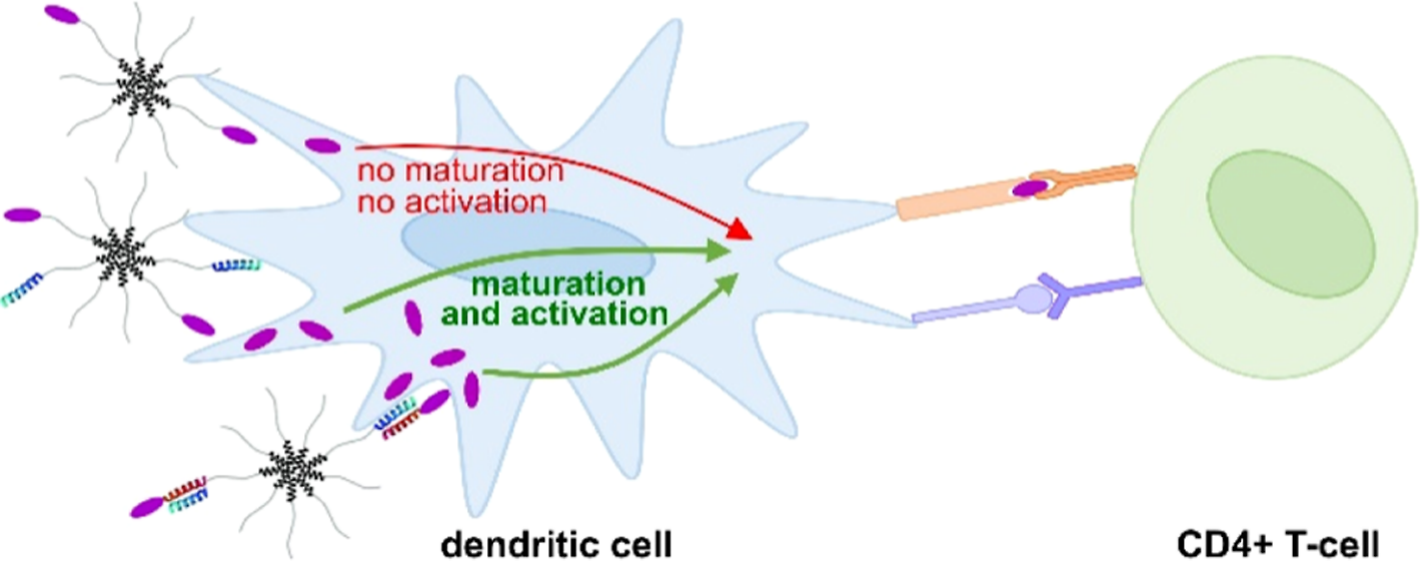

Subunit vaccines would benefit from a safe particle-based adjuvant. Elastin-like polypeptide (ELP)-based micelles are interesting candidate adjuvants due to their well-defined size and easy modification with protein-based cargo. Coiled coils can facilitate noncovalent modifications, while potentially enhancing antigen delivery through interaction with cell membranes. ELP micelles comprise ELP diblock copolymers that self-assemble above a critical micelle temperature. In this study, an amphiphilic ELP was conjugated to peptide “K”, which forms a heterodimeric coiled-coil complex with peptide “E”. Self-assembled “covalent” micelles containing ELP-OVA323 (i.e., model antigen OVA323 conjugated to ELP), “coiled-coil” micelles containing ELP-K/E-OVA323 and “hybrid” micelles containing ELP-K and ELP-OVA323 were shown to be monodisperse and spherical. Dendritic cells (DCs) were exposed to all micelle compositions, and T-cell proliferation was investigated. The presence of ELP-K enhanced micelle uptake and subsequent DC maturation, resulting in enhanced CD4^+^ T-cell proliferation, which makes ELPs with coiled coil-associated antigens a promising vaccine platform.

## Introduction

Vaccination is an effective method to prevent infectious diseases and decrease the associated mortality.^1^ Traditional vaccination strategies are based on live attenuated or inactivated pathogens. These conventional vaccines are linked to adverse effects, such as the risk of infection in immunocompromised recipients or excessive immune responses to the various inflammatory elements that inactivated or live attenuated vaccines contain.^2^ In the search for safer vaccines, research has increasingly focused on subunit vaccines, which contain antigenic components of pathogens and omit unnecessary elements. Subunit vaccines may be safer than traditional vaccines; however, this comes at the cost of lower efficacy. Since subunit vaccines generally do not generate the same level of immunity, they require an adjuvant.^2,3^ The most commonly used adjuvant is alum, which refers to micrometer-sized particles based on aluminum salts. However, injections with an alum can cause local adverse effects^4^ and are also associated with some systemic side effects.^5,6^ Moreover, alum is unsuitable for inducing immunity against intracellular targets such as mycobacteria (e,g. *Mycobacterium tuberculosis*) and parasites (e.g., *Plasmodium falciparum*) because it primarily triggers a strong humoral response.^7^ Thus, there is a need for safe, particle-based adjuvants that induce a strong cellular immune response.

Multiple factors are important for the design of effective adjuvants. Good adjuvants increase the uptake of the antigen into antigen-presenting cells (APCs), such as dendritic cells (DCs), and promote subsequent DC maturation. This involves the development of additional dendrites and expressing major histocompatibility complex II (MHCII) and costimulatory molecules such as CD80 and CD86.^8^ These costimulatory molecules are presented on the DC surface and can interact with CD28 on CD4^+^ T-cells in secondary lymphoid organs, thereby stimulating T-cells to expand and differentiate.^9,10^

Interestingly, studies have shown that the adjuvant effect is more pronounced if antigens are physically attached to the adjuvant when taken up by DCs.^11−13^ Moreover, immune stimulatory peptide sequences can also enhance the potency of nanoparticle-based adjuvants to modulate or increase the immune response.^14^ For instance, coiled coil-forming peptides exhibit immune-stimulating activity, depending on their primary amino acid sequence.^15−17^ Coiled coils are noncovalent complexes composed of interacting α-helices forming a supercoil, and the assembly is driven by specific hydrophobic and electrostatic interactions. Their adjuvant effect is caused by increased antigen uptake by cells through peptide–membrane interactions.^18−22^ For example, proteins with a coiled-coil domain modified with a specific cationic peptide induced an increased immune response to the conjugated antigens.^15^ We recently showed that polymer-based nanoparticles displaying coiled-coil peptide K at the surface increased the immunogenicity of an influenza antigen.^16,17^ Peptide K is a class A amphiphilic helix peptide, which is known to interact with and/or destabilize membranes.^23^ Peptide K binds parallel to membranes,^24^ and due to its cationic and amphiphilic properties, it may have cell-penetrating peptide (CPP) activity.^25^ Alternatively, coiled-coil domains can interact with scavenger receptors from DCs.^26^ Both membrane binding and CPP characteristics potentially increase the uptake of coiled coils and conjugates thereof into cells. For example, a higher level of exposure to a hexameric coiled-coil complex increased the level of endocytosis for conjugated IgG antibodies.^19^ Furthermore, the coiled-coil domains in polydnavirus^20^ and *Salmonella enterica*(21) are necessary for targeting cell membranes. Moreover, Bode et al. used a heterodimeric coiled-coil complex to combine two tetra-arginine segments to create a functional CPP, exhibiting a 4-fold higher uptake as compared to the equivalent octa-arginine peptide.^22^

In this study, elastin-like polypeptide (ELP) diblock copolymer micelles are explored as an adjuvant for vaccine delivery. ELPs are temperature-responsive polypeptides composed of repeating VPGXG pentapeptides in which guest residue X can be any amino acid. Amphiphilic ELP diblock copolymers with different guest residues for each block assemble into spherical micelles above a critical micelle temperature (CMT). The small size and spherical shape of these ELP micelles are well suited for inducing a strong CD4^+^ T-cell response.^27^ Moreover, these micelles can be easily functionalized with peptide or protein antigens by standard recombinant DNA methods. The resulting micelles are decorated covalently with antigens at the periphery like spike proteins on coronaviruses. However, the requirement for intracellular processing of covalently conjugated antigens can negatively influence the desired immune response.^28^ In contrast, coiled coil-mediated conjugation is reversible and could release the potential protein. Moreover, the coiled-coil complex is stable, ensuring colocalization, but dissociates at low pH, enabling endosomal escape.^29^ This article evaluates whether the immune response is dependent on the method of antigen conjugation to the nanoparticle adjuvant. Covalent antigen attachment is compared to attachment via a heterodimeric coiled-coil complex. We previously studied the heterodimeric coiled-coil pair consisting of peptide E ((EIAALEK)_*n*_) and peptide K ((KIAALKE)_*n*_). The dissociation constant of the E_3_/K_3_ coiled-coil complex is low (*K*_d_ = 73 nM),^30^ making this heterodimeric coiled-coil complex suitable to bind antigens to adjuvant nanoparticles in a noncovalent yet stable fashion.

The coiled coil-mediated association of antigens to adjuvants is of interest for three reasons. The first is to study the relation between the strength of antigen attachment to the adjuvant and the induced immune response by examining the difference between covalent and noncovalent attachment strategies. Furthermore, each strategy has implications for internal processing and the resulting ability to correctly present the OVA323 epitopes on MHCII. Lastly, the presence of coiled-coil domains may increase the immune response due to its tendency to interact with cellular membranes.

**Figure 1:**
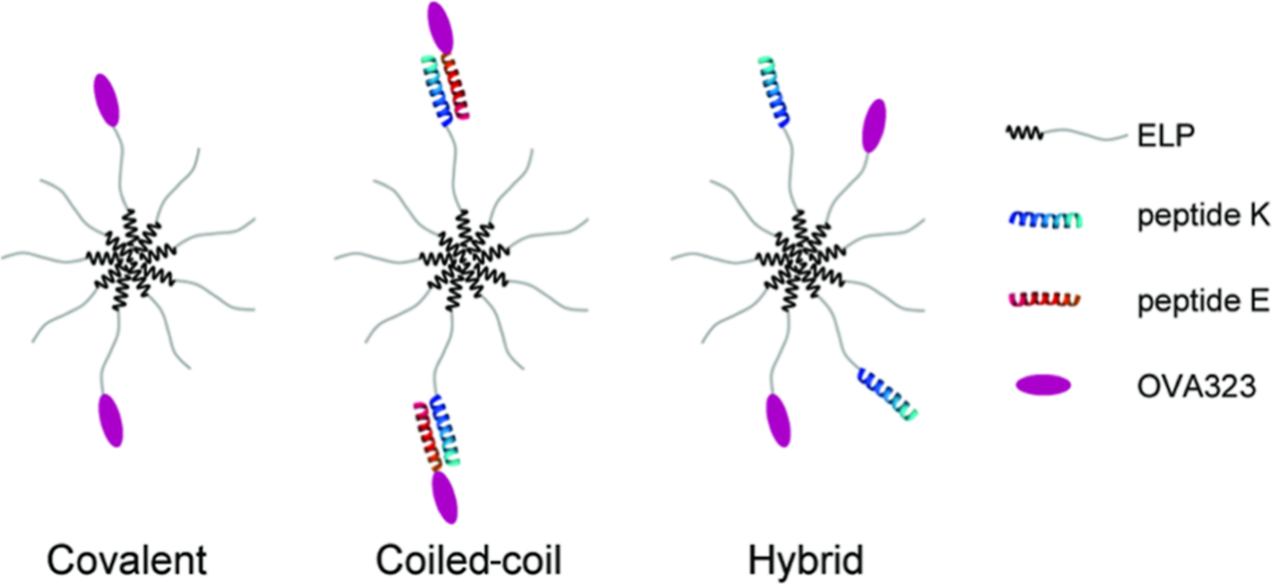
Micellar architectures displaying the OVA323 used in this study. All formulations are constructed on ELP micelles. The covalent micelle contains ELP/ELP-OVA323 [9:1]. The coiled-coil micelles link the antigen to the micelle noncovalently in the following molar ratio of ELP/ELP-K/E-OVA323 [9:1:1]. A hybrid micelle controls for the effects of peptide K without a binding partner, which includes ELP/ELP-OVA323/ELP-K [8:1:1]. Cartoon is not to scale.

The peptide antigen “OVA323” (amino acids 323–339 from ovalbumin) was chosen as a model antigen to study the immunogenicity of the ELP adjuvants. We designed three differently functionalized micelles, called “covalent”, “coiled-coil”, and “hybrid” (Figure 1), referring to the conjugation strategy of OVA323 to ELP. Since the coiled-coil and covalent micelles differ both in the strength of attachment and in the presence of peptide K on the micelle corona, hybrid micelles were designed to evaluate the independent effect of ELP-K on the covalently linked peptide antigen.

The amount of antigen cargo per micelle can be controlled by changing the ratio of plain ELP to functionalized ELP, enabling optimization of the desired immune response. In this study, the covalent, coiled-coil, and hybrid formulations are compared to nonfunctionalized micelles (ELP) and fully functionalized micelles (ELP-OVA323).

First, the effect of functionalization with the coiled-coil peptide K on the ELP micelles was studied. The sequences of all used polypeptides and peptides are listed in Table 1. Static light scattering (SLS) was used to determine the CMT and the critical micelle concentration (CMC), while the hydrodynamic diameter of the micelles as a function of composition was measured by dynamic light scattering (DLS). Micelle morphology was investigated using atomic force microscopy (AFM) and transmission electron microscopy (TEM). Subsequently, micelle uptake in bone marrow-derived DCs (BMDCs) was studied using flow cytometry and confocal microscopy. Flow cytometry analysis of these BMDCs also included quantification of DC maturation and CD4^+^ T-cell stimulation.

**Table 1:** Names and Sequences of Polypeptides and Peptides Used in This Study^a^

name	sequence
ELP	MG(VPGIG)_48_(VPGSG)_48_Y
ELP-K	MG(VPGIG)_48_(VPGSG)_48_YWSGGG(KIAALKE)_4_
ELP-OVA323	MG(VPGIG)_48_(VPGSG)_48_YGISQAVHAAHAEINEAGR
E	⟨Ac⟩-YG(EIAALEK)_3_-⟨NH_2_⟩
OVA323	YGISQAVHAAHAEINEAGR
E-OVA323	⟨Ac⟩-YG(EIAALEK)_3_ISQAVHAAHAEINEAGR-⟨NH_2_⟩
E-OVA323-GC	⟨Ac⟩-YG(EIAALEK)_3_ISQAVHAAHAEINEAGRGC-⟨NH_2_⟩
E-OVA323-TMR	⟨Ac⟩-YG(EIAALEK)_3_ISQAVHAAHAEINEAGRGC ⟨TMR⟩-⟨NH_2_⟩

a Sequences are given in one-letter amino acid abbreviations. ⟨Ac⟩ = Acetyl, ⟨NH_2_⟩ = amide, ⟨TMR⟩ = tetramethyl rhodamine, covalently linked to the cysteine by a thio-ether bond.

## Materials and Methods

### Chemicals and Reagents

Ultrapure water was obtained using a Milli-Q system. Phosphate buffer (PB) contained 10 mM potassium phosphate (pH 7.8). Phosphate buffered saline (PBS) contained 10 mM PB potassium phosphate (pH 7.6) and 150 mM NaCl. Cells were cultured in Iscove’s Modified Dulbecco’s Medium (IMDM). FACS buffer contained PBS with 1% FCS and 2 mM EDTA.

### Plasmids

A pET25 expression vector coding for ELP was provided by the MacKay laboratory.^31^ The XbaI-AcuI (blunted by T4 DNA polymerase treatment) DNA fragment comprising the ELP sequence was recloned into XbaI-*Sma*I digested pET52b(+) and maintained in *Escherichia coli* XL10-Gold. In the resulting pET52b-ELP plasmid, XbaI and BseRI sites are available for inserting DNA fragments upstream, and Acc65I, *Bam*HI, BsrGI, *Sal*I, EagI, NotI, SacI, and AvrII sites are available for inserting DNA fragments downstream of the ELP coding sequence. The plasmids encoding ELP-K and ELP-OVA323 were constructed by cloning the respective DNA fragments (BaseClear, Leiden, The Netherlands) into the Acc651 and NotI sites.

### Polypeptide Expression and Purification

ELP, ELP-K, and ELP-OVA323 were expressed by transforming the plasmids into *E. coli* BL21(DE3) cells using the heat shock and calcium methods.^32^ The cell culture was grown in an LB medium containing ampicillin (250 μg/mL) at 37 °C. A starter culture of 10 mL was added to 1 L of media, and the cells were cultured until an OD600 of ∼0.5. The cultures were cooled to 18 °C and induced overnight with 0.05 mM IPTG. The cells were harvested and washed with 0.9% NaCl solution, and the cell pellet was frozen at −80 °C. Cells were lysed in PB containing 1 mM pefablock (Roche Diagnostics), 1 mg/mL lysozyme (Thermo Scientific)), and 2 mM MgCl_2_, 25 u/mL benzonase (250 u/μL; Sigma-Aldrich) in a total volume of 10 mL. The mixture was incubated at 4 °C for approximately 45 min and sonicated with a 13 mm probe on ice at 25% amplitude for 5 min in 5 s intervals. The solids were removed from the lysate by centrifugation at 4 °C (37,000 rpm, 228,783 rcf) for 30 min. The ELPs were purified by a round of inverse transition cycling. NaCl was added to the lysate to reach a concentration of 4 M. After incubation for 30 min at room temperature, the mixture was centrifuged (10,000 rpm, 17,100 rcf) for 30 min. The pellet was suspended in cold PB and incubated for at least 30 min at 4 °C. After centrifugation (10,000 rpm, 17,100 rcf) for 30 min at 4 °C, the soluble ELP was collected by decanting the supernatant. Four additional cycles of ELP-mediated purification were applied, using 3 M NaCl for the incubation at room temperature. The final supernatant was dialyzed against PB. The ELP concentration was determined using UV–vis spectroscopy. ELP was frozen in liquid N_2_ and then stored at −20 °C.

### FITC Labeling of ELP

To a solution of ELP (276 mM) in 0.1 M NaHCO_3_ pH 9 was added 8 equiv of fluorescein isothiocyanate (FITC) from a 5 mg/mL stock in DMSO. The mixture was light-protected and incubated overnight at 4 °C. The reaction was quenched with 0.5 M NH_4_Cl (25 equiv with respect to the dye) for 2 h in the dark at 4 °C. Excess dye was removed on a PD-10 desalting column (8.3 mL bed volume, GE Healthcare) according to the manufacturer’s instructions. The concentration was determined using the initial polypeptide concentration corrected for the dilution during the reaction and purification. The labeling factor was determined using UV–vis spectroscopy. Labeled ELPs were frozen in liquid N_2_ and then stored at −20 °C.

### Peptide Synthesis and Purification

Peptide E was synthesized on a Biotage Syro I fully automated parallel peptide synthesizer using standard Fmoc chemistry. Rink amide resin with a loading of 0.55 mmol/g (Sigma-Aldrich) was used as a support. Coupling reactions were performed with 0.5 M HCTU (Novabiochem) in DMF (Biosolve), 2 M DIPEA (Carl Roth) in a 1:1 mixture of NMP (Biosolve) and DMF, and 0.5 M Fmoc-protected amino acid (Novabiochem) in DMF. Deprotection steps were carried out with 40% piperidine (Biosolve) in DMF. All solutions contained 1 g/L LiCl (Sigma-Aldrich). Up from the 15th coupling step, amino acids were coupled to the peptide using double coupling steps. The N-terminus was acetylated with 0.5 M acetic anhydride (Biosolve) and 0.125 M DIPEA in NMP for 2 h. The peptide was cleaved from the resin using a mixture of 2.5% triisopropylsilane (Sigma-Aldrich), 2.5% water, and 95% TFA (Biosolve) and subsequently precipitated in cold diethyl ether (Honeywell). The precipitate was collected by centrifugation, dissolved in water, and lyophilized. The peptide was purified by preparative reverse phase HPLC and the purity was confirmed with LC–MS (Figures S1–S2).

E-OVA323 and E-OVA323-GC were synthesized on a CEM Liberty Blue automated peptide synthesizer using similar methods to those described above.

### Fluorescent Labeling of E-OVA323-GC

1.85 mg of E-OVA323-GC (0.42 μmol) was dissolved in 2.07 mL of PBS containing 10 mM EDTA and 1 mM TCEP. One mg of TMR maleimide (21 μmol) in another 2.07 mL of buffer was added, and the mixture was incubated at room temperature in the dark for 2 h. Excess dye was removed using a centrifugal spin column (2 kDa MWCO, Sartorius Vivaspin) and peptide E-OVA323-TMR was purified by reverse phase HPLC. Successful labeling of the peptide was confirmed with LC–MS (Figure S3).

### UV–Vis Spectroscopy

Polypeptide concentrations were determined based on the absorbance at 280 nm. The absorption was measured on an Agilent 8453 device at 10 °C. The theoretical extinction coefficients were calculated using the ProtParam tool of ExPasy: ε_ELP_ and ε_ELP-OVA_ = 1490 L mol^–1^ cm^–1^; ε_ELP-K_ = 6990 L mol^–1^ cm^–1^.^33^ The labeling factor (number of dye molecules per polypeptide molecule) was determined by measuring the absorption at 494 nm for FITC-ELP (ε_FITC_ of 70,000 L mol^–1^ cm^–1^).

### DLS and SLS

All SLS and DLS measurements were performed on a Malvern Zetasizer Nano-S instrument using BRAND UV cuvettes micro. The sample (75 μL) was equilibrated to the desired temperature for 3.5 min. Three measurements were performed, each consisting of 12 runs of 10 s and averaged. For DLS measurements and SLS measurements used to determine the CMT, the attenuator was automatically set by Zetasizer software. The CMCs were determined by dilution of a stock solution to various concentrations. Each of these diluted samples was measured with SLS, with the attenuator set to 11. The count rates were normalized to the count rate of PB and plotted as a function of log [polypeptide]. The shape of the autocorrelation functions was used to determine whether particles were detected. One trendline was fitted through the data points for which particles were detected and another through those of nonparticulate ELPs. The CMC was determined as the concentration corresponding to the intercept of these trendlines.

### Zeta Potential

Zeta potential measurements were performed on a Malvern Zetasizer Nano-ZS using a Malvern Zetasizer nano series Universal Dip Cell kit. One mL of a 2.5 μM polypeptide solution in PB was incubated at 37 °C before starting the measurements.

### Atomic Force Microscopy

Samples for AFM were prepared by drop-casting 20 μL of 37 °C 2 μM polypeptide on a silicon oxide wafer (Siegert Wafer) with a 285 nm thermal oxide layer or on a mica disc (V1 grade; Muscovite). The samples were dried at 37 °C for 30 min. AFM images were recorded using a JPK NanoWizard Ultra Speed microscope, and the obtained data were processed using the JPK SPM Data Processing software. All experiments were performed using a silicon probe (Olympus, OMCL-AC160TS) with a nominal resonance frequency of 300 kHz. The images were all scanned and recorded (with a resolution of 512 × 512 pixels) in intermittent contact mode in air at room temperature.

### BMDCs

BMDCs were cultured as described in the literature.^34^ Bone marrow was taken from the hind legs of wild-type C57BL/6 and TIM4^–/–^ mice. The bone marrow cells were suspended using a 70 μm cell strainer (Greiner Bio-One). The cells were incubated for 10 days at 37 °C and 5% CO_2_ in IMDM (Lonza) containing 2 mM  l-glutamine, 8% (v/v) FCS, 100 U/mL penicillin/streptomycin (Lonza), 50 μM β-mercaptoethanol (Sigma), and 20 ng/mL GM-CSF (PeproTech). The medium was refreshed every 2–3 days. For the first 9 days, the cells were cultured in 95 mm Petri dishes (Greiner Bio-One) and for the last day, in 96 well F-bottom plates (Greiner Bio-One).

### DC Uptake and Maturation

Fluorescently labeled micelles in PB were diluted 10 times with media and added to the DCs in 96 well plates (for flow cytometry analysis) and an 8 well plate (Ibidi GmbH; for confocal microscopy). Hoechst dye (final concentration of 0.01 mg/mL) was added to each of the wells of the 8 well plate. Each well contained 50,000 cells in 200 μL. For the negative control, BMDCs were treated with media only. The cells were incubated at 37 °C with 5% CO_2_ for 4 h. The cells attached to the bottom of the 8 well plate were washed with media ten times and imaged by confocal microscopy. The cells in the 96 well plates were isolated by centrifugation and transferred with 100 μL of 4 mM EDTA to the U-bottom 96 well plates. Excess EDTA was removed by centrifugation, and the cells were labeled with live/dead APC-Cy7, CD11cPeCy7, and CD86APC (Invitrogen) in FACS buffer for 20 min at 4 °C. Next, the cells were spun down, washed with FACS buffer, spun down again, resuspended in FACS buffer, and analyzed by flow cytometry.

### T-Cell Isolation

T-cells were obtained as described in the literature.^35^ Spleens from donor mice were mashed with a syringe plunger and suspended in PBS through a 70 μm cell strainer. The buffer was removed by centrifugation and the red blood cells were lysed for 1 min with 0.15 M NH_4_Cl, 1 mM KHCO_3_, and 0.1 mM Na_2_EDTA (pH 7.3). CD4^+^ T-cells were isolated by negative selection using sheep-antirat IgG Dynabeads (Dynal, Invitrogen) and an excess amount of anti-B220 (RA3–6B2), anti-CD11b (M1/70), anti-MHCII (M5/114), and anti-CD8 (YTS169) mAb in magnetic-activated cell sorting (MACS; Miltenyi Biotec) buffer. The cells were spun down and resuspended in 1 μM carboxyfluorescein diacetate succinimidyl ester (CFSE; Molecular Probes) in PBS. Next, the cells were labeled for 10 min at 37 °C and 5% CO_2_ before removing excess CFSE by centrifugation. A suspension of 250,000 cells/mL in media was prepared and stored at 4 °C.

### T-Cell Activation

Various micelle solutions and peptide solutions of the peptides of the micellars of OVA323 (Invivogen) or E-OVA323 in PB were diluted 10 times with media and added to the DCs in the 96-well plates. Each well contained 10,000 cells. OVA323 concentrations were 270, 90, 30, 10, 3.3, and 1.1 nM, which correspond to total polypeptide concentrations of 2.7 μM, 900, 300, 100, 33, and 11 nM, respectively. The control samples (ELP, ELP/ELP-K, and ELP/OVA323) were included only at a total polypeptide concentration of 2.7 μM. As a negative control, BMDCs were treated with media only. Cells were incubated at 37 °C with 5% CO_2_ for 4 h and centrifuged. After the removal of the supernatant containing the micelles/peptides, a suspension of 50,000 T-cells in media was added to the DCs, and the cells were mixed. Next, the cells were incubated for 3 days at 37 °C in 5% CO_2_. After centrifugation, the cells were resuspended in FACS buffer containing CD4efluor, Thy1.2PeCy7, CD25APC, CD67Pe, and live/dead APC-Cy7 (Invitrogen). After incubation at 4 °C for 20 min, the buffer and excess antibodies were removed by centrifugation and the cells were taken up in FACS buffer. The cells were analyzed by flow cytometry.

### Flow Cytometry

Flow cytometry was performed on a CytoFLEX S Beckman Coulter device. Analysis of the data was performed using FlowJo software.

### Confocal Microscopy

BMDCs were visualized on an SP8 LIGHTNING Confocal Microscope using a 63x lens. The colocalization percentage was determined using ImageJ software by setting the lower and upper thresholds at 15 and 255, respectively, and subsequently running the 3D MultiColoc plugin of the 3D Image suite.

### Statistics

Flow cytometry data were analyzed in Graphpad Prism 8 for Windows. Groups were compared with an ordinary one-way ANOVA and Tukey’s multiple comparison test.

## Results and Discussion

### Expression and Purification of ELPs

ELP consists of a hydrophobic block at the N-terminus and a hydrophilic block at the C-terminus, facilitating assembly into well-defined micelles.^31^ ELP, ELP-K, and ELP-OVA323 were expressed in *E. coli* and purified with five cycles of inverse transition cycling (Figure S4). Polypeptide yields were 41 mg/L (ELP), 38 mg/L (ELP-K), and 55 mg/L (ELP-OVA323), respectively, in line with previous reports.^31,36^ The exact mass of each polypeptide was verified by ESI-TOF mass spectrometry (Figure S4) and the purity (>95%) by HPLC (Figure S5).

### Self-Assembly Behavior

**Figure 2:**
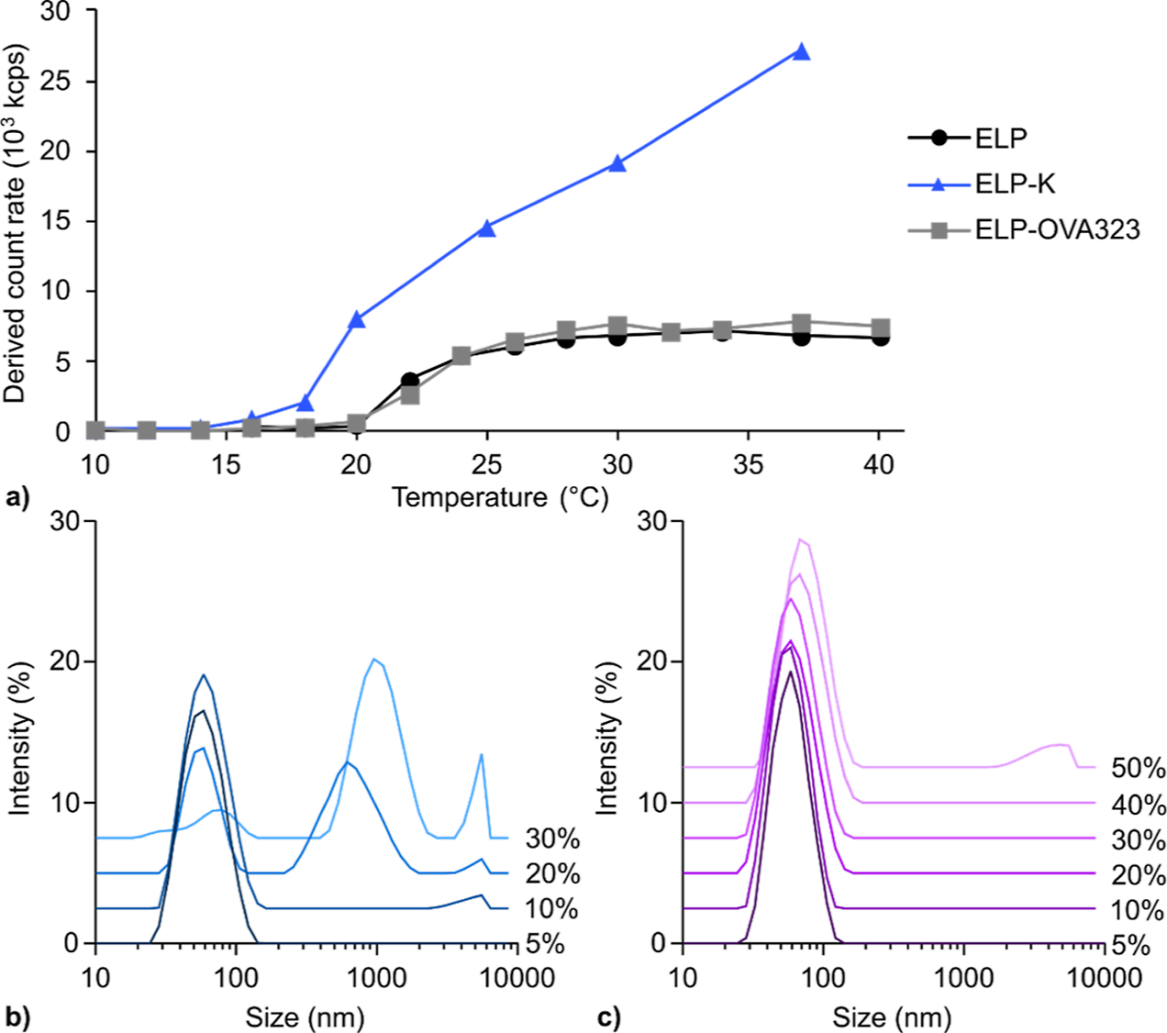
Self-assembly of ELP, ELP-K, and ELP-OVA323 micelles. (a) The CMT for micelle assembly was detected by SLS at a concentration of 10 μM in 10 mM PB (pH 7.8) as a function of temperature. Above the assembly temperature of ∼24 °C, ELP and ELP-OVA323 maintained stable count rates. In contrast, ELP-K alone did not form a stable micelle. (b,c) size distributions of ELP/ELP-K micelles containing various percentages of ELP-K in the absence (b) and presence (c) of equimolar amounts of peptide E. [polypeptide] = 20 μM; E/K ratio = 1; *T* = 37 °C; measured in 10 mM PB, pH 7.8. *Z*-average values are included in Table S1.

The inverse transition behavior of the polypeptides was studied with SLS measurements to analyze the effect of extending ELPs with peptide K or OVA323 on self-assembly in solution. The ELPs were dissolved in phosphate buffer (PB) and light scattering was recorded as a function of temperature. ELP-K assembly was already observed at a lower temperature compared to ELP and ELP-OVA323 (Figure 2a). The critical micelle or aggregation temperature (CMT or CAT) is defined as the temperature at which the polypeptide starts to aggregate, resulting in an increase in scattering. This transition occurred at 22 °C for ELP and ELP-OVA323 and at 16 °C for ELP-K. While ELP and ELP-OVA323 were assembled into micelles, ELP-K formed large aggregates (Table 2). Peptide K is prone to homodimerization and is also known to interact with membranes, burying the hydrophobic face of the helix in the bilayer while the lysine side chains “snorkel” toward the polar/apolar interface.^24^ Therefore, it is possible that peptide K also has an affinity for the hydrophobic domain of ELP micelles. This might result in intramicellar and intermicellar interactions between peptide K and the ELP core, inducing severe aggregation. This would possibly explain the continuing growth of the count rate with an increasing temperature.

**Table 2:** Physicochemical Properties of Micelles^a^

formulation^b^	*D*_h_ [nm]^c^	PdI^d^	zeta potential [mV]
ELP	53.7 ± 0.7	0.033 ± 0.011	–4.3 ± 0.3
ELP-K	1266 ± 291	0.244 ± 0.059	n.d.
10% ELP-K (ELP/ELP-K [9:1])	60.8 ± 1.0	0.192 ± 0.017	–4.5 ± 0.5
ELP-OVA323	54.5 ± 0.3	0.031 ± 0.015	–7.3 ± 1.5
covalent (ELP/ELP-OVA323 [9:1])	55.7 ± 0.9	0.080 ± 0.012	–4.7 ± 0.7
coiled-coil (ELP/ELP-K/E-OVA323 [9:1:1])	57.5 ± 0.6	0.114 ± 0.011	–4.3 ± 0.2
hybrid (ELP/ELP-K/ELP-OVA323 [8:1:1])	56.8 ± 0.1	0.109 ± 0.023	–4.0 ± 0.2

a [polypeptide] = 10 μM for DLS and 2.5 μM for zeta potential; measured in 10 mM PB, pH 7.8, at 37 °C. Size distributions are shown in Figure S8. Values represent average values ± SD (*n* = 3).

b Ratios indicated for the formulations are molar ratios.

c Hydrodynamic diameter.

d Polydispersity index. N.d. = not determined.

ELP-K aggregation could be avoided by mixing this polypeptide with plain ELP. ELP/ELP-K (9:1) mixtures formed stable micelles at 37 °C with a hydrodynamic diameter comparable to plain ELP micelles (54 and 61 nm, respectively). However, increasing the percentage of ELP-K induced significant aggregation (Figure 2b). Interestingly, the equimolar addition of peptide E in the mixtures prevented aggregation of up to 40% ELP-K (Figure 2c). The presence of peptide E prevents aggregation of ELP/ELP-K micelles most likely because heterodimer coiled-coil formation is favored over peptide K homodimerization. These results show that it is possible to formulate stable ELP/ELP-K mixed micelles, as long as peptide E is added before micelle assembly. The DLS measurements in Figure 2 were performed for a duration of approximately 10 min, but ELP-based micelles are typically stable for >12 h at 37 °C (Figure S6).

ELP/ELP-K (9:1) micelles were somewhat larger and more polydisperse compared with plain ELP micelles (Table 2). TEM revealed the formation of spherical micelles with comparable size distributions for all formulations (Figure S7). The zeta potential was near neutral for all formulations due to the absence of charged amino acids in ELP, the major component in these assemblies.

Next, the critical aggregation concentration (CAC) of ELP-K and the CMC of ELP and ELP-OVA323 were determined using SLS (Figures S9–S10). The CAC of ELP-K was considerably lower (56 nM) than the CMC of ELP (150 nM), confirming the strong influence of peptide K on the ELP assembly.

In contrast to the extension of ELP with peptide K, conjugation of OVA323 did not affect the inverse transition behavior, as demonstrated by the similar CMT, CMC, size, and surface charge (Table 2) of ELP-OVA323 compared to ELP micelles. This is because the OVA323 epitope contains only 17 residues, is fully water-soluble, and is not known to oligomerize.

### Properties of OVA323 Displaying Micelles

**Figure 3:**
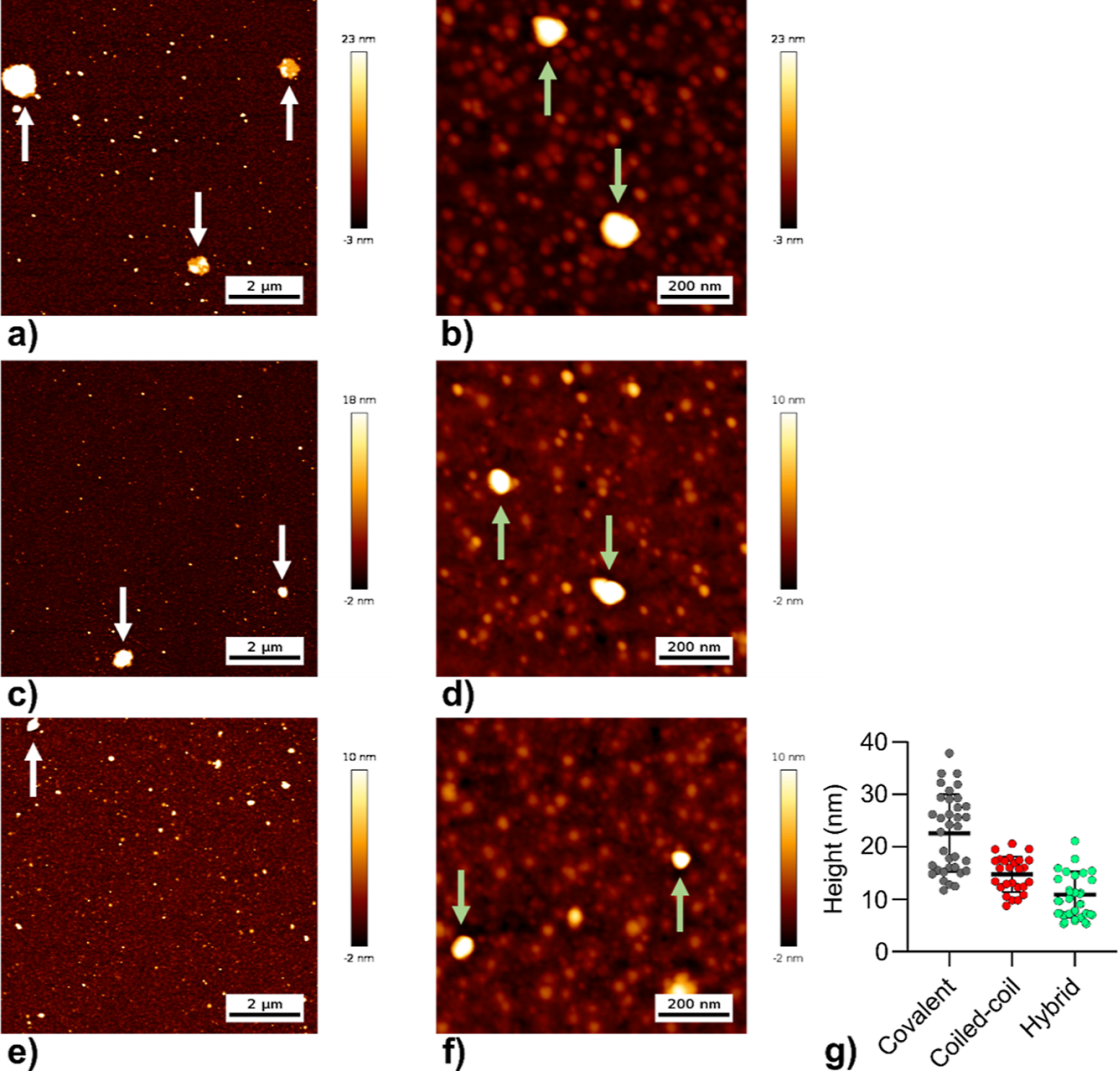
AFM images of covalent (a,b), coiled-coil (c,d), and hybrid (e,f) micelles. [polypeptide] = 2 μM in water, deposited on a silicon oxide surface and dried at 37 °C; height trace mode; imaged using medium (a,c,e) and high (b,d,f) magnifications. The medium magnifications were used to analyze the average height of the imaged micelles (g): 23 nm (covalent), 15 nm (coiled-coil), and 11 nm (hybrid). Micelles are indicated with green arrows and clustered micelles are indicated with white arrows. Each sample was also imaged using error trace mode (Figure S11), at low magnification (Figure S12), and on a mica surface (Figure S13).

Covalent, coiled-coil, and hybrid micelles (Figure 1) were prepared by mixing the individual polypeptide and peptide solutions in the correct ratio and heating the resulting formulations to 37 °C. The resulting assemblies were analyzed with DLS and zeta potential measurements revealing comparable size and surface charge of these micelles, albeit with a higher polydispersity compared to ELP or ELP-OVA323 micelles (Table 2). Even under a low ionic strength, the surface charge of each micelle formulation was near neutral. This was expected as each formulation contains charge-neutral ELP as the main component by mass. Next, the morphology of the micelles was investigated with AFM (Figures 3; S11–S13) and TEM (Figure S14).

AFM studies showed that the covalent, coiled-coil, and hybrid samples were all assembled into spherical particles with an average height of 23, 15, and 11 nm, respectively (Figure 3g). These observed sizes are much smaller than the hydrodynamic diameter observed with DLS (∼57 nm). Typically, hydrodynamic diameters are larger than imaged diameters by AFM.^37^ For this technique, a sample of these dynamic micelles is dried on a silicon oxide surface, which might result in the flattening of the assemblies. This effect might be more pronounced for the coiled-coil and hybrid micelles, explaining the measured particle height differences. The observed large assemblies are most likely clustered micelles, as a result of sample preparation. TEM imaging confirmed the size range (∼10 to 50 nm) observed with AFM and DLS (Figure S14).

Coiled-coil formation on the micelle corona was proven using coiled-coil micelles mixed with tetramethyl rhodamine (TMR) labeled E-OVA323 (E/K ratio = 1). These micelles were thoroughly washed and subsequently, the remaining E-OVA323 peptide was quantified (Figure S15). A 3-fold higher amount of E-OVA323 remained on coiled-coil micelles compared to ELP micelles, confirming that E-OVA323 is conjugated to the micelles via E/K coiled-coil formation. This is in line with previous studies concerning coiled coil-mediated antigen conjugation to the surface of liposomes.^38,39^

### ELP-K Facilitates Uptake of Micelles in DCs

Next, the effect of displaying OVA323 at the surface of covalent, coiled-coil, and hybrid micelles on the association and uptake of APCs was investigated. BMDCs were exposed to micelle formulations, each containing 8% FITC-labeled ELP (FITC-ELP). To examine the potential immune-stimulating role of peptide K in BMDC uptake, ELP/ELP-K [9:1] micelles were also included in this study. In addition, the coiled-coil sample contained TMR-labeled E-OVA323 (15% labeled). The use of different fluorescent dyes for E-OVA323 and ELP enabled us to study the antigen uptake independently of the micelle uptake. Free E-OVA323-TMR peptide was included as a control for micelle-independent uptake. First, BMDCs were incubated with various formulations and analyzed with flow cytometry, which showed that micelle uptake is concentration dependent up from 100 nM polypeptide concentrations (Figure S16), which is close to the CMCs (i.e., ranging from 56 to 150 nM, see Figure S9). None of the samples at any tested concentration had an apparent impact on cell viability as none of the samples caused concentration-dependent dehydrogenase (LDH) release by the cells (Figure S17). Moreover, the BMDCs had a similar appearance between groups and controls, as well as between sample concentrations within groups when observed by light microscopy (data not shown).

**Figure 4:**
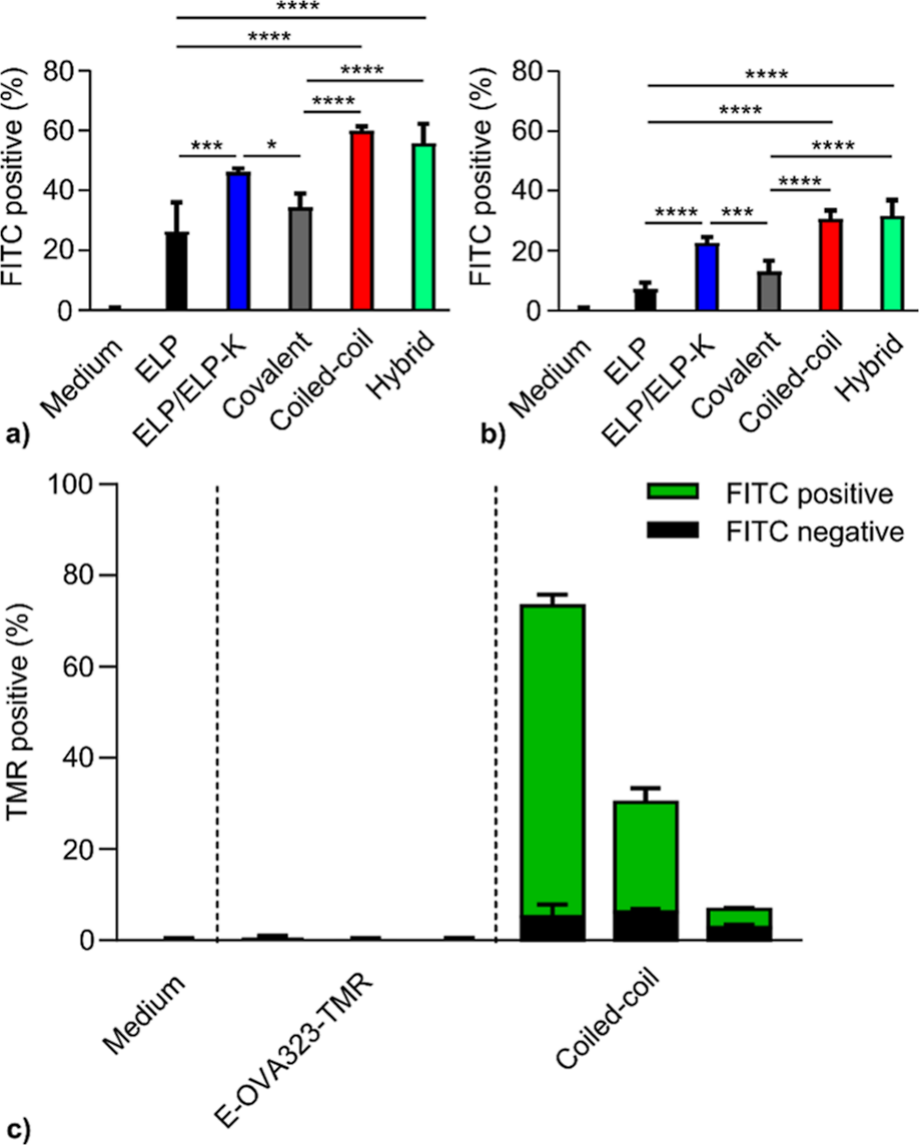
Flow Cytometry reveals that ELP micelles enhance the association of OVA323 antigens with BMDCs. Cells were incubated for 4 h with fluorescently labeled micelles (FITC), OVA peptide (TMR), or with media alone as a negative control. After washing, BMDCs were analyzed by flow cytometry according to the gating strategy shown in Figure S18 (a,b) Percentage of BMDCs positive for association (e.g., binding and/or uptake). of FITC-labeled micelles containing 90 nM (a) or 30 nM (b) OVA323, corresponding to 900 nM and 300 nM polypeptide, respectively. Significant differences between BMDC uptake percentages of samples with ELP-K and samples without ELP-K were determined using a one-way ANOVA with a Tukey’s multiple comparison test. * = *p* < 0.05, ** = *p* < 0.01, *** = *p* < 0.001, **** = *p* < 0.0001. (c) Percentage of BMDC positive for uptake of the micelle (FITC) and coiled coil-associated antigen (TMR). [OVA323] (from left to right) = 270, 90, and 30 nM. Mean ± SD.

Using flow cytometry, all micelle formulations containing ELP-K (i.e., ELP/ELP-K, coiled-coil, and hybrid) showed higher cellular association compared to micelle formulations without ELP-K (i.e., ELP and covalent), see Figure 4a,b. Peptide K likely binds to the plasma membrane of BMDCs,^24^ forcing the micelles in close proximity to the cell surface, which is a prerequisite to cellular uptake and antigen presentation. To evaluate the specificity of this association, dual-color flow cytometry was used to examine if cells positive for the FITC-labeled coiled-coil micelles were also positive for TMR-labeled E-OVA323 (Figure 4c). While E-OVA323 alone did not associate with many BMDCs, its formulation with ELP-K micelles via a coiled-coil assembly ensured efficient cellular association of the antigen. Most of the E-OVA323 (i.e., TMR signal)-positive cells were also ELP (i.e., FITC signal) positive: 93%, 80%, and 59% for 270, 90, and 30 nM E-OVA323, respectively. These double-positive cells confirm successful micelle-dependent association with cells, whereas a small fraction of TMR-positive, FITC-negative cells appeared to associate with the antigen without associating with the ELP carrier.

**Figure 5:**
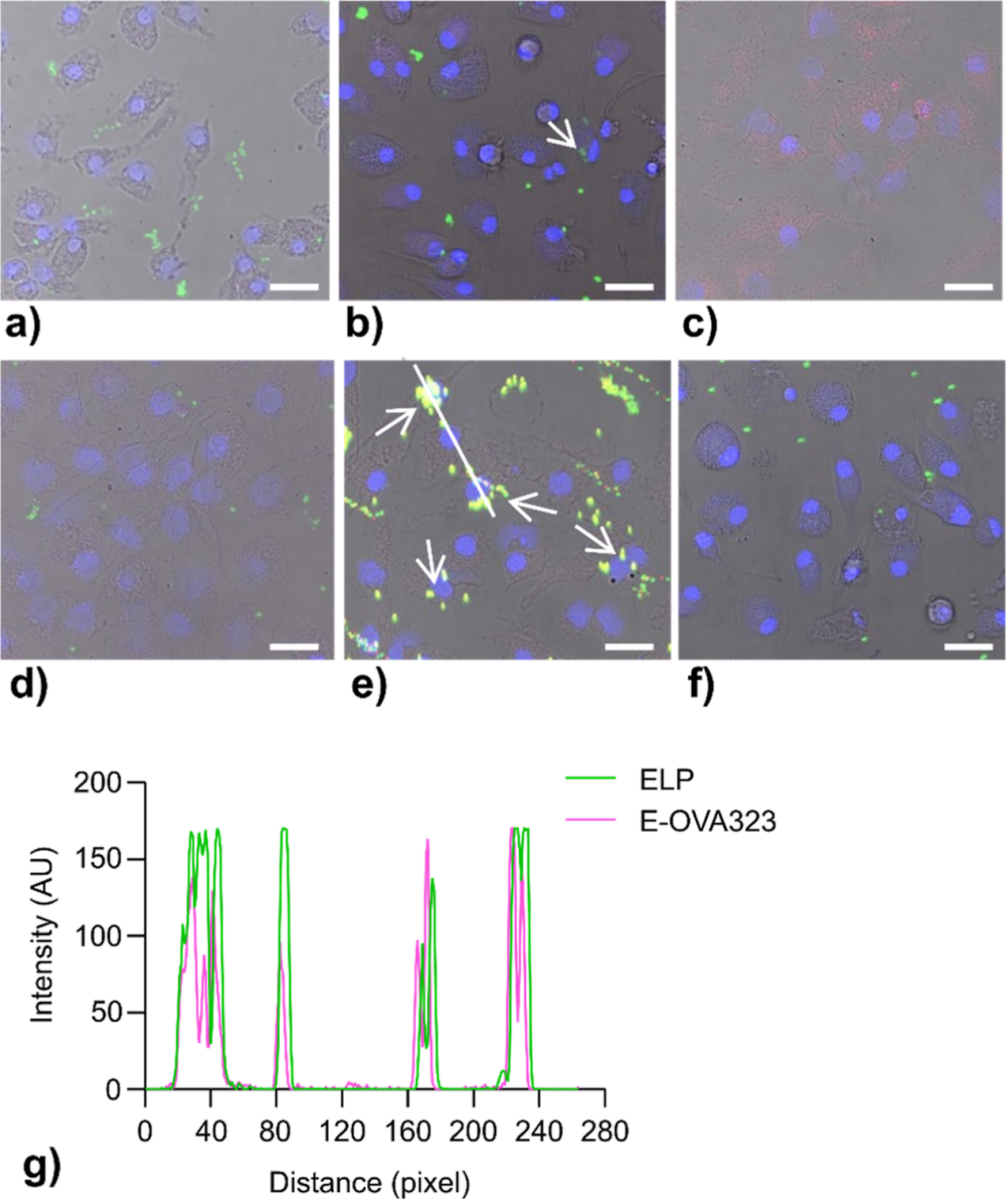
Confocal microscopy shows the ELP-K-dependent colocalization of coiled-coil micelles into BMDCs. Cells were incubated for 4 h with fluorescently labeled peptide (TMR) or micelles (FITC) containing 90 nM OVA323 (900 nM ELP). BMDCs were washed with media to remove excess particles and imaged on a confocal microscope. Images were combined from each sample: bright-field image, blue channel (cell nuclei), green channel (ELP), and red channel (E-OVA323). (a–f) Overlays of the fluorescent Z-stacks with the bright field image are shown for each sample: (a) ELP; (b) ELP/ELP-K; (c) E-OVA323; (d) covalent; (e) coiled-coil; and (f) hybrid. Separate channels and three-dimensional images of cells that have taken up fluorescent material are compiled in Figure S19. While E-OVA323 alone shows diffuse staining in most cells, the coiled-coil formulation revealed perinuclear puncta, which is consistent with endolysosomal trafficking. As indicated by arrows (e), their yellow color suggests the colocalization of ELP and E-OVA323. In fact, 94.4% of the E-OVA323 signal (red) in this image is colocalized with the ELP signal (green). Along the white line (e), the intensities of the ELP and E-OVA323 signals appear spatially correlated, which also is consistent with the couptake of ELP and E-OVA323 (g). Scale bar: 25 μM.

Having demonstrated that the OVA323 antigen associates with DCs better in formulation with ELP micelles, live-cell confocal laser scanning microscopy was used to assess the degree to which antigens were internalized after 4 h of incubation in complete media (Figure 5). After extensive washes with IMDM media, little cell uptake was observed for ELP micelles or covalent micelles. Those micelles may not have been internalized but were merely adhered to the outside of a cell membrane. Thus, plain ELP micelles do not effectively enter the BMDCs at this concentration. In contrast, micelles containing ELP-K (i.e., ELP/ELP-K, coiled-coil, and hybrid) were internalized efficiently inside cells. These results indicate that ELP-K is essential for BMDC uptake, presumably due to its interaction with cell membranes.^24^ Confocal imaging also confirmed that the free E-OVA323 peptide is not readily taken up by BMDCs. However, E-OVA323 conjugated to ELP-K containing micelles via coiled-coil formation was taken up efficiently (Figure 5e), which is consistent with the flow cytometry (Figure 4c). Furthermore, BMDCs pulsed with coiled-coil micelles revealed colocalization of ELP and E-OVA323, confirming the stability of the coiled-coil complex upon cell uptake (Figure 5g).

### Peptide-K Induces DC Maturation

**Figure 6:**
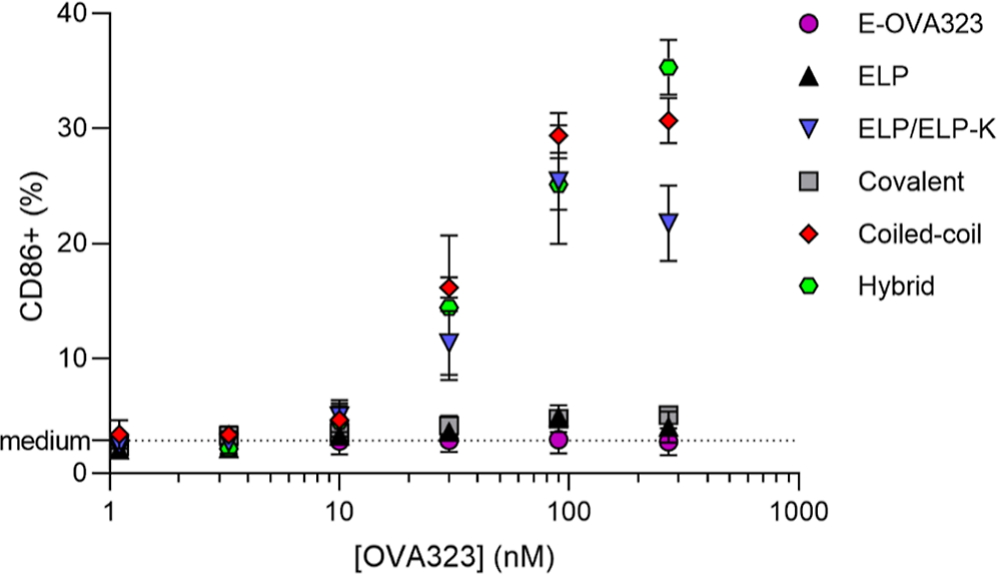
Maturation of BMDCs following exposure to ELP-K-containing micelles. BMDCs were incubated for 4 h with micelles or with E-OVA323 peptide or media only as negative controls. OVA323 concentrations in the E-OVA323 peptide, covalent micelle, coiled-coil micelle, and hybrid micelle formulations were 1.1, 3.3, 10, 30, 90, and 270 nM, corresponding to 11, 33, 100, 300, 900, and 2700 nM polypeptide. The plain ELP micelles and ELP/ELP-K micelles were added in the same polypeptide concentrations as the other samples. The BMDCs were subsequently analyzed by flow cytometry. Representative CD86 histogram plots are shown in Figure S20. Means of CD86 positive BMDC percentages (*n* = 4) are plotted, and the error bars depict the standard deviations.

After antigen uptake, DC maturation is the next step to effectively stimulate the T helper cells. Therefore, the expression of the costimulatory molecule CD86, a marker for DC maturation, was quantified. ELP-K containing micelle formulations induced more CD86 expression above the CMC (ranging from 56 to 150 nM polypeptide, see Figure S9) as compared to micelles without this polypeptide (Figure 6). This shows that peptide K may stimulate the immune response by inducing both increased maturation and more efficient uptake of antigens. Peptide K is known to interact with membranes and potentially induces membrane disruption,^24^ which could in turn result in enhanced BMDC maturation.^40^

### T-Cell Proliferation by ELP Micelles Displaying OVA323

After antigen uptake and subsequent maturation, DCs can induce antigen-specific T-cell proliferation. Therefore, CD4^+^ T-cells were isolated from OT-II transgenic mice, which exclusively contained T-cells with OVA323-specific T-cell receptors. These T-cells were labeled with carboxyfluorescein diacetate N-succinimidyl ester (CFSE) and cocultured with BMDCs previously exposed to the micelle formulations. Finally, subsequent T-cell proliferation was quantified with flow cytometry, by tracking the dilution of the CFSE signal.

**Figure 7:**
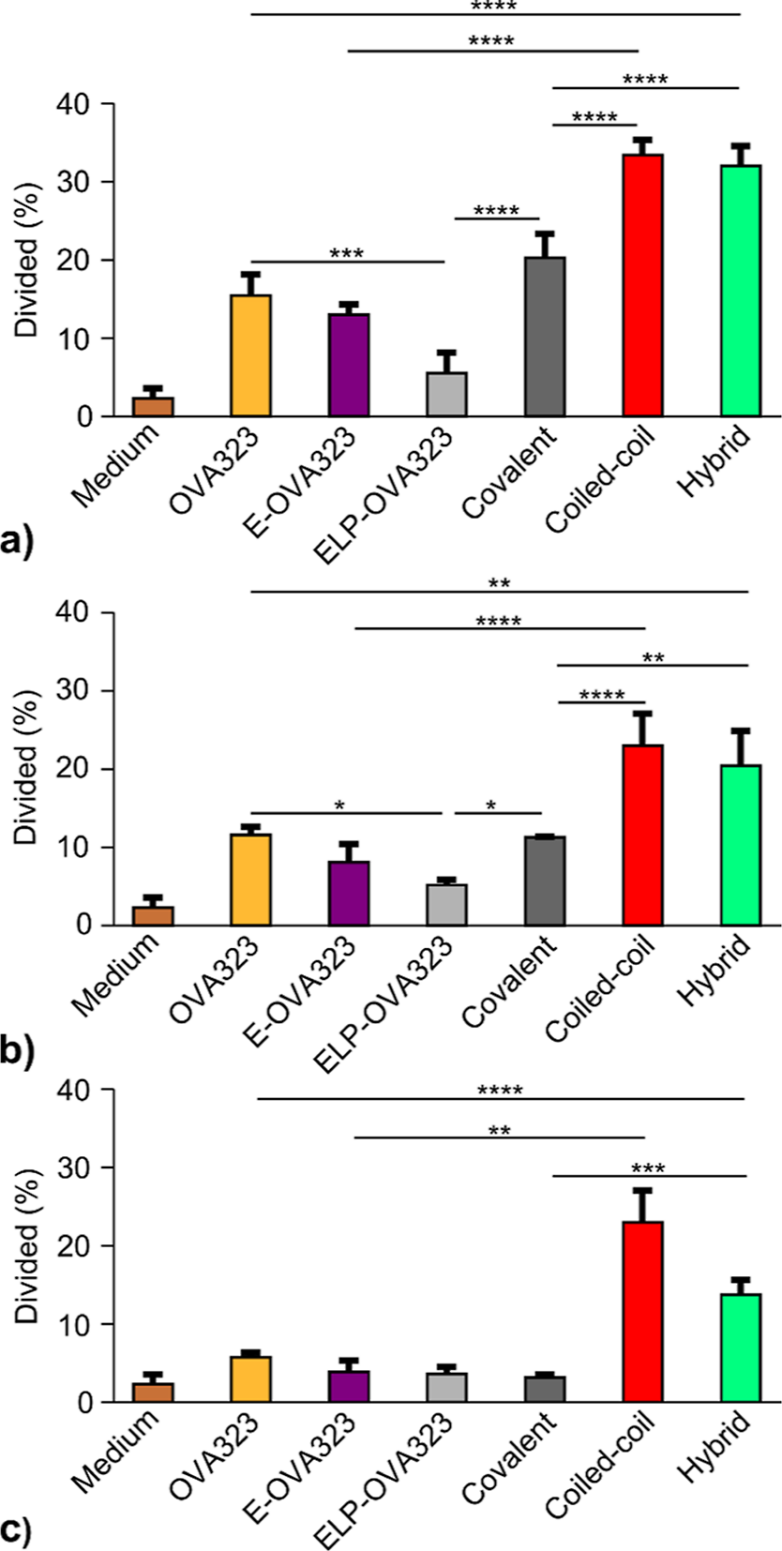
Proliferation of OT-II cells in vitro. [OVA323]: 270 (a), 90 (b), and 30 nM (c), corresponding to 2700, 900, and 300 nM polypeptide, respectively. BMDCs were incubated for 4 h with micelles, peptides, or with media only. The BMDCs were subsequently exposed for 3 days to OT-II cells containing fluorescent dye. The decrease in fluorescent signal in the OT-II population was then analyzed with flow cytometry. Representative examples of CFSE dilution plots are shown in Figure S22. The bars depict the mean and standard deviation of divided cells. Significant differences between divided cell percentages were determined using a one-way ANOVA with Tukey’s multiple comparison test. * = *p* < 0.05, ** = *p* < 0.01, *** = *p* < 0.001, **** = *p* < 0.0001.

Besides the covalent, coiled-coil, and hybrid micelles, additional controls were included in this study. OVA323 peptide, E-OVA323 peptide, ELP-OVA323 micelles, and a mixture of ELP micelles with free OVA323 peptide were also evaluated. ELP micelles, ELP/ELP-K micelles, and cell media were used as negative controls. As expected, T-cell proliferation was dependent on the concentration of OVA323, and ELP micelles without OVA323 did not induce T-cell proliferation (Figure S21). Interestingly, ELP-OVA323 did not induce OT-II proliferation at any of the tested concentrations (Figure 7).

Potentially, the covalent conjugation of ELP to the OVA323 epitope had a strong negative effect on T-cell growth, most likely due to the need for internal processing before the epitope can be presented on MHCII. In contrast, E-OVA323, which also requires internal processing, induced a level of T-cell proliferation as OVA323. This study reveals that the internal processing of the antigen is sensitive to the conjugation method (i.e., covalent vs coiled-coil). Interestingly, treatment of BMDCs with ELP-OVA323 micelles also resulted in less divided T-cells compared to those in the covalent micelle group. Since the covalent group comprises more micelles per antigen, this result indicates that ELP micelles function as an adjuvant. The lower antigen loading per micelle was also favorable. The covalent micelles had an effect on T-cell proliferation similar to that of free OVA323. The positive influence of increased uptake by the micelles may have compensated for the negative influence of antigen processing issues related to covalently attaching the ELP to OVA323. The coiled-coil and hybrid groups did have significantly stronger effects on T-cell proliferation compared to those of their free peptide counterparts E-OVA323 and OVA323, respectively. These groups also outperformed the covalent group, suggesting that the presence of peptide K provides an additional advantage over plain ELP micelles. Taken together, these results indicate that the presence of ELP-K stimulates the division of T-cells. This is likely caused by the increased uptake of ELP-K-containing formulations, as well as the higher level of DC maturation. Therefore, ELP-K may be a valuable asset to increase the efficacy of ELP micelle-based drug delivery vehicles such as the “ELP/ELP-Bet v 1” birch pollen allergen immunotherapy platform.^41,42^ In those works, the birch pollen allergen was evaluated in vivo to demonstrate the functional relevance of the ELP micelle presentation strategy with an allergen that is relevant to human immunity. Instead, this work focuses on the optimization of the presentation strategy for the model antigen, OVA323. While the coiled-coil formulation has the most promising in vitro behavior, future studies will be needed to reveal which formulation strategies (covalent, coiled-coil, and hybrid) are optimal in vivo.

## Conclusions

In this study, the potential of ELP micelles in vaccine development was explored as both an antigen carrier and an adjuvant for inducing a cellular immune response. Using the model antigen OVA323, two methods of antigen conjugation were explored, covalent versus noncovalent conjugation. For the latter approach, the highly specific and stable heterodimeric coiled-coil peptide pair “E” (EIAALEK)_3_ and “K” (KIAALKE)_4_ was used. Covalent OVA323-carrying micelles were composed of ELP/ELP-OVA323 (ratio of 9:1), while coiled-coil micelles contained ELP/ELP-K/EOVA323 (ratio of 9:1:1). Due to its amphipathic nature, peptide K interacts with cell membranes and can therefore act as an adjuvant. To examine the effect of ELP-K on the immune response independently from the effect of the conjugation method, “hybrid” (ELP/ELP-K/ELP-OVA, ratio of 8:1:1) micelles were also included in this study. DLS and AFM studies showed that all formulations assembled into spherical and monodisperse nanoparticles. Remarkably, the presence of ELP-K increased BMDC uptake and maturation as well as enhanced proliferation of CD4^+^ T-cells. Furthermore, covalently conjugated OVA323 epitopes induced less CD4^+^ T-cell proliferation when compared to that of soluble OVA323. In contrast, peptide E conjugation to OVA323 did not affect the proliferation of CD4^+^ T-cells, suggesting that coiled coil-mediated conjugation does not complicate cellular antigen processing. In summary, ELP micelles displaying peptide K stimulate an antigen-specific cellular immune response.

## Supporting Information Available

Experimental methods for preparative RP-HPLC, LC–MS, SDS-PAGE, TEM, mass spectrometry, assessment of coiled-coil association efficiency, fluorescence spectroscopy, LDH assay, and LPS assay; size and dispersity of micelles; CMC/CAC determinations; AFM and TEM images of micelles; BMDC uptake of FITC-labeled micelles; LDH activity of ELP micelles; confocal images of BMDCs; proliferation of OT-II cells cocultured with BMDCs (PDF)

## References

[ref1] Orenstein W. A.; Ahmed R. Simply Put: Vaccination Saves Lives. Proc. Natl. Acad. Sci. U.S.A. 2017, 114 (16), 4031–4033. 10.1073/pnas.1704507114. 28396427 PMC5402432

[ref2] Cao Y.; Zhu X.; Kakar P.; Zhao Y.; Chen X. Augmentation of Vaccine-Induced Humoral and Cellular Immunity by a Physical Radiofrequency Adjuvant. Nat. Commun. 2018, 9, 3695 10.1038/s41467-018-06151-y. 30209303 PMC6135850

[ref3] Zhang R.; Kramer J. S.; Smith J. D.; Allen B. N.; Leeper C. N.; Li X.; Morton L. D.; Gallazzi F.; Ulery B. D. Vaccine Adjuvant Incorporation Strategy Dictates Peptide Amphiphile Micelle Immunostimulatory Capacity. AAPS J. 2018, 20, 73 10.1208/s12248-018-0233-6. 29858738

[ref4] Zhao Z.; Ukidve A.; Krishnan V.; Mitragotri S. Effect of Physicochemical and Surface Properties on in Vivo Fate of Drug Nanocarriers. Adv. Drug Delivery Rev. 2019, 143, 3–21. 10.1016/j.addr.2019.01.002. 30639257

[ref5] HogenEsch H. Mechanism of Immunopotentiation and Safety of Aluminum Adjuvants. Front. Immunol. 2013, 3 (JAN), 1–13. 10.3389/fimmu.2012.00406. PMC3541479 23335921

[ref6] Vogelbruch M.; Nuss B.; Körner M.; Kapp A.; Kiehl P.; Bohm W. Aluminium-Induced Granulomas after Inaccurate Intradermal Hyposensitization Injections of Aluminium-Adsorbed Depot Preparations. Allergy 2000, 55, 883–887. 10.1034/j.1398-9995.2000.00501.x. 11003454

[ref7] Gregory A. E.; Titball R.; Williamson D. Vaccine Delivery Using Nanoparticles. Front. Cell. Infect. Microbiol. 2013, 3 (March), 1–13. 10.3389/fcimb.2013.00013. 23532930 PMC3607064

[ref8] Kim M. K.; Kim J. Properties of Immature and Mature Dendritic Cells: Phenotype, Morphology, Phagocytosis, and Migration. RSC Adv. 2019, 9, 11230–11238. 10.1039/c9ra00818g. 35520256 PMC9063012

[ref9] Sun B.; Xia T. Nanomaterial-Based Vaccine Adjuvants. J. Mater. Chem. B 2016, 4 (33), 5496–5509. 10.1039/c6tb01131d. 30774955 PMC6377210

[ref10] Kaech S. M.; Wherry E. J.; Ahmed R. Effector and Memory T-Cell Differentiation: Implications for Vaccine Development. Nat. Rev. Immunol. 2002, 2 (April), 251–262. 10.1038/nri778. 12001996

[ref11] Slütter B.; Soema P. C.; Ding Z.; Verheul R.; Hennink W.; Jiskoot W. Conjugation of Ovalbumin to Trimethyl Chitosan Improves Immunogenicity of the Antigen. J. Controlled Release 2010, 143 (2), 207–214. 10.1016/j.jconrel.2010.01.007. 20074597

[ref12] Slütter B.; Bal S. M.; Que I.; Kaijzel E.; Löwik C.; Bouwstra J.; Jiskoot W. Antigen-Adjuvant Nanoconjugates for Nasal Vaccination: An Improvement over the Use of Nanoparticles?. Mol. Pharm. 2010, 7 (6), 2207–2215. 10.1021/mp100210g. 21043518

[ref13] Wang Z.; Xu J. Better Adjuvants for Better Vaccines: Progress in Adjuvant Delivery Systems, Modifications, and Adjuvant-Antigen Codelivery. Vaccines 2020, 8 (1), 128–220. 10.3390/vaccines8010128. 32183209 PMC7157724

[ref14] Ni Q.; Zhang F.; Liu Y.; Wang Z.; Yu G.; Liang B.; Niu G.; Su T.; Zhu G.; Lu G.; Zhang L.; Chen X. A Bi-Adjuvant Nanovaccine That Potentiates Immunogenicity of Neoantigen for Combination Immunotherapy of Colorectal Cancer. Sci. Adv. 2020, 6 (12), 1–12. 10.1126/sciadv.aaw6071. PMC7080439 32206706

[ref15] del Campo Ascarateil J.; Turki Hani I.; Hill F. Modified Coiled Coil Type Proteins Having Improved Properties. U.S. Patent 9,388,225 B2, 2016.

[ref16] Barnier Quer C.; Robson Marsden H.; Romeijn S.; Zope H.; Kros A.; Jiskoot W. Polymersomes Enhance the Immunogenicity of Influenza Subunit Vaccine. Polym. Chem. 2011, 2 (7), 1482–1485. 10.1039/c1py00010a.

[ref17] Zope H.; Quer C. B.; Bomans P. H. H.; Sommerdijk N. A. J. M.; Kros A.; Jiskoot W. Peptide Amphiphile Nanoparticles Enhance the Immune Response against a CpG-Adjuvanted Influenza Antigen. Adv. Healthcare Mater. 2014, 3 (3), 343–348. 10.1002/adhm.201300247. 23983195

[ref18] Apostolovic B.; Deacon S. P. E.; Duncan R.; Klok H. A. Cell Uptake and Trafficking Behavior of Non-Covalent, Coiled-Coil Based Polymer-Drug Conjugates. Macromol. Rapid Commun. 2011, 32 (1), 11–18. 10.1002/marc.201000434. 21432965

[ref19] Dhankher A.; Lv W.; Studstill W. T.; Champion J. A. Coiled Coil Exposure and Histidine Tags Drive Function of an Intracellular Protein Drug Carrier. J. Controlled Release 2021, 339 (March), 248–258. 10.1016/j.jconrel.2021.09.026. PMC8599652 34563592

[ref20] Asgari S.; Schmidt O. A Coiled-Coil Region of an Insect Immune Suppressor Protein Is Involved in Binding and Uptake by Hemocytes. Insect Biochem. Mol. Biol. 2002, 32 (5), 497–504. 10.1016/S0965-1748(01)00127-8. 11891126

[ref21] Knodler L. A.; Ibarra J. A.; Pérez-Rueda E.; Yip C. K.; Steele-Mortimer O. Coiled-Coil Domains Enhance the Membrane Association of Salmonella Type III Effectors. Cell. Microbiol. 2011, 13 (10), 1497–1517. 10.1111/j.1462-5822.2011.01635.x. 21679290 PMC3418822

[ref22] Bode S. A.; Kruis I. C.; Adams H. P. J. H. M.; Boelens W. C.; Pruijn G. J. M.; van Hest J. C. M.; Löwik D. W. P. M. Coiled-Coil-Mediated Activation of Oligoarginine Cell-Penetrating Peptides. ChemBioChem 2017, 18, 185–188. 10.1002/cbic.201600614. 27870530

[ref23] Segrest J. P.; de Loof H.; Dohlman J. G.; Brouillette C. G.; Anantharamaiah G. M. Amphipathic Helix Motif: Classes and Properties. Proteins 1990, 8, 103–117. 10.1002/prot.340080202. 2235991

[ref24] Rabe M.; Schwieger C.; Zope H. R.; Versluis F.; Kros A. Membrane Interactions of Fusogenic Coiled-Coil Peptides: Implications for Lipopeptide Mediated Vesicle Fusion. Langmuir 2014, 30 (26), 7724–7735. 10.1021/la500987c. 24914996

[ref25] Hansen M.; Kilk K.; Langel Ü. Predicting Cell-Penetrating Peptides. Adv. Drug Delivery Rev. 2008, 60, 572–579. 10.1016/j.addr.2007.09.003. 18045726

[ref26] McKenzie E. J.; Mukhopadhyay S.; Gordon S.; Martinez-Pomares L. Scavenger Receptors on Dendritic Cells. In Handbook of Dendritic Cells: Biology, Diseases, and Therapies; WILEY-VCH Verlag GmbH & Co. KGaA: Weinheim, 2006; pp 141–163.

[ref27] Benne N.; van Duijn J.; Kuiper J.; Jiskoot W.; Slütter B. Orchestrating Immune Responses: How Size, Shape and Rigidity Affect the Immunogenicity of Particulate Vaccines. J. Controlled Release 2016, 234, 124–134. 10.1016/j.jconrel.2016.05.033. 27221070

[ref28] Kramer K.; Shields N. J.; Poppe V.; Young S. L.; Walker G. F. Intracellular Cleavable CpG Oligodeoxynucleotide-Antigen Conjugate Enhances Anti-Tumor Immunity. Mol. Ther. 2017, 25 (1), 62–70. 10.1016/j.ymthe.2016.10.001. 28129129 PMC5363295

[ref29] Wu Y.; Collier J. H. α-Helical Coiled-Coil Peptide Materials for Biomedical Applications. Wiley Interdiscip. Rev.: Nanomed. Nanobiotechnol. 2017, 9 (2), e1424 10.1002/wnan.1424. PMC5300935 27597649

[ref30] Crone N. S. A.; Kros A.; Boyle A. L. Modulation of Coiled-Coil Binding Strength and Fusogenicity through Peptide Stapling. Bioconjugate Chem. 2020, 31 (3), 834–843. 10.1021/acs.bioconjchem.0c00009. PMC7086394 32058706

[ref31] Janib S. M.; Pastuszka M.; Aluri S.; Folchman-Wagner Z.; Hsueh P.-Y.; Shi P.; Lin Y. A.; Cui H.; Mackay J. A. A Quantitative Recipe for Engineering Protein Polymer Nanoparticles. Polym. Chem. 2014, 5 (5), 1614–1625. 10.1039/c3py00537b. 24511327 PMC3916011

[ref32] Chang A. Y.; Chau V. W. Y.; Landas J. A.; Pang Y. Preparation of Calcium Competent Escherichia Coli and Heat-Shock Transformation. JEMI Methods 2017, 1, 22–25.

[ref33] ProtParam tool. https://web.expasy.org/protparam/ (accessed 12 21, 2021).

[ref34] Benne N.; van Duijn J.; Lozano Vigario F.; Leboux R. J. T.; van Veelen P.; Kuiper J.; Jiskoot W.; Slütter B. Anionic 1,2-Distearoyl-Sn-Glycero-3-Phosphoglycerol (DSPG) Liposomes Induce Antigen-Specific Regulatory T Cells and Prevent Atherosclerosis in Mice. J. Controlled Release 2018, 291 (October), 135–146. 10.1016/j.jconrel.2018.10.028. 30365993

[ref35] Keijzer C.; Slütter B.; van der Zee R.; Jiskoot W.; van Eden W.; Broere F. PLGA, PLGA-TMC and TMC-TPP Nanoparticles Differentially Modulate the Outcome of Nasal Vaccination by Inducing Tolerance or Enhancing Humoral Immunity. PLoS One 2011, 6 (11), e26684 10.1371/journal.pone.0026684. 22073184 PMC3206834

[ref36] Pille J.; Van Lith S. A. M.; Van Hest J. C. M.; Leenders W. P. J. Self-Assembling VHH-Elastin-like Peptides for Photodynamic Nanomedicine. Biomacromolecules 2017, 18 (4), 1302–1310. 10.1021/acs.biomac.7b00064. 28269985 PMC5388898

[ref37] Domingos R. F.; Baalousha M.; Ju-nam Y.; Reid M. M.; Tufenkji N.; Lead J. R.; Leppard G. G.; Wilkinson K. J. Characterizing Manufactured Nanoparticles in the Environment: Multimethod Determination of Particle Sizes. Environ. Sci. Technol. 2009, 43, 7277–7284. 10.1021/es900249m. 19848134

[ref38] Leboux R. J. T.; Benne N.; van Os W. L.; Bussmann J.; Kros A.; Jiskoot W.; Slütter B. High-Affinity Antigen Association to Cationic Liposomes via Coiled Coil-Forming Peptides Induces a Strong Antigen-Specific CD4+ T-Cell Response. Eur. J. Pharm. Biopharm. 2021, 158, 96–105. 10.1016/j.ejpb.2020.11.005. 33188929

[ref39] Warmenhoven H.; Leboux R.; Bethanis A.; van Strien J.; Logiantara A.; van Schijndel H.; Aglas L.; van Rijt L.; Slütter B.; Kros A.; Jiskoot W.; van Ree R. Cationic Liposomes Bearing Bet v 1 by Coiled Coil-Formation Are Hypo-Allergenic and Induce Strong Immunogenicity in Mice. Front. Allergy 2023, 3, 1–13. 10.3389/falgy.2022.1092262. PMC9872006 36704756

[ref40] Nace G.; Evankovich J.; Eid R.; Tsung A. Dendritic Cells and Damage-Associated Molecular Patterns: Endogenous Danger Signals Linking Innate and Adaptive Immunity. J. Innate Immun. 2011, 4 (1), 6–15. 10.1159/000334245. 22086146

[ref41] van Strien J.; Escalona-Rayo O.; Jiskoot W.; Slütter B.; Kros A. Elastin-like Polypeptide-Based Micelles as a Promising Platform in Nanomedicine. J. Controlled Release 2023, 353 (December 2022), 713–726. 10.1016/j.jconrel.2022.12.033. 36526018

[ref42] van Strien J.; Warmenhoven H. J. M.; Logiantara A.; Makurat M.; Aglas L.; Bethanis A.; Leboux R. J. T.; van Rijt L. S.; MacKay J. A.; van Schijndel J. W. P. M.; Schneider G. F.; Olsthoorn R. C. L.; Jiskoot W.; van Ree R.; Kros A. Bet v 1-Displaying Elastin-like Polypeptide Nanoparticles Induce a Strong Humoral and Weak CD4+ T-Cell Response against Bet v 1 in a Murine Immunogenicity Model. Front. Immunol. 2022, 13 (October), 1–15. 10.3389/fimmu.2022.1006776. PMC9583423 36275650

