# Supplementary material for: Noncovalent Conjugation of OVA323 to ELP Micelles Increases Immune Response
Source: Biomacromolecules. 2024 Jan 3;25(2):1027–37. doi: 10.1021/acs.biomac.3c01091 (PMC10865353; doi:10.1021/acs.biomac.3c01091)
Supplement: Supplementary file 1 — bm3c01091_si_001.pdf [file bm3c01091_si_001.pdf]

Non-covalent conjugation of OVA323 to ELP micelles increases immune response

*Jolinde van Strien, Max Makurat, Ye Zeng, René R. C. L. Olsthoorn, Gregory F. Schneider, Bram A. Slütter, J. Andrew MacKay, Wim Jiskoot, Alexander Kros **

SUPPORTING INFORMATION

Supplemental methods

Preparative RP-HPLC

Peptides were purified using preparative reverse phase HPLC on a Shimadzu system consisting of two LC-8A pumps and an SPD-10AVP UV–Vis detector, and equipped with a Kinetex Evo C18 column. The following gradients of acetonitrile (Biosolve) in water, both containing 0.1% TFA, were used: 10% to 35% followed by a plateau at 35% for peptide E, 10% to 80% for peptide K and 20% to 80% for E-OVA323, E-OVA323-GC and E-OVA323-TMR. The purity of the fractions was confirmed by LC-MS. Pure fractions were combined and lyophilized to obtain the peptide as a powder. The purity and identity of each peptide was confirmed with LC-MS (Figures S20-22).

LC-MS

LC-MS was measured on a Thermo Scientific TSQ access MAX device for mass detection combined with an Ultimate 3000 liquid chromatography system. Liquid chromatography was performed using a gradient of 0 to 90% acetonitrile in water containing 0.1% TFA on a 50 × 4.6 mm Phenomenex Gemini 3 µm C18 column.

SDS-PAGE

Samples for SDS-PAGE were mixed with reducing Laemmli sample buffer and, without heating first, loaded on a 10% SDS poly acrylamide gel. Electrophoresis was performed at 200 V. The gel used to analyze the purification process of ELP-K was stained with Coomassie Brilliant Blue R-250 (Bio-Rad). The ELP and ELP-OVA323 gels were stained with 0.5 M $CuCl_2$ for 15 minutes, washed with water for 3 x 5 minutes, and the stained gels were photographed on a black background.

TEM

A 20 µM solution of polypeptide in water, water and 1% uranyl acetate (each 10 µL) were placed on a piece of parafilm and incubated at 37 °C for 2 minutes. A preheated copper grid was subsequently placed on top of the polypeptide solution, water and staining droplets, every time removing excess liquid by blotting with filter paper. The grid was incubated at 37 °C for 1-2 hours and imaged on a JEOL TEM 1010 electron microscope with an accelerating voltage of 100 kV.

Mass spectrometry

The mass of ELP, FITC-ELP and TRITC-ELP was determined by ESI-QTOF on a Nanoacquity UPLC system (Waters) connected to a Synapt G2Si mass spectrometer (Waters) using an Acquity UPLC M-Class column (300 µm x 50 mm), packed with BEH C4 material (particle diameter = 1.7 µm; pore size = 300 Å). 5 µL sample was injected and the components were separated using a gradient of 10 to 90% acetonitrile (Biosolve) in water, both containing 0.1% formic acid (Sigma Aldrich). Electro-spray ionization (ESI) was used *via* Nano-spray source with ESI emitters (New Objectives) fused silica tubing 360 µm OD x 25 µm ID tapered to 5 ± 0.5 µm (5 nL/cm void volume). The following settings in positive resolution mode were used: source temperature of 80 °C, capillary voltage 4.5 kV, nano flow gas of 0.25 bar, purge gas 250 L/h, trap gas flow 2.0 mL/min, cone gas 100 L/h, sampling cone 25 V, source offset 25, trap CE 32 V, scan time 3.0 seconds, mass range 400-2400 m/z. Lock mass acquiring was performed with a mixture of Leu Enk (556.2771) and Glu Fib (785.84265), lockspray voltage 3.5 kV, Glufib fragmentation was used as calibrant. Masslynx software was used for acquisition and Ent3 software for polymer

envelope signal deconvolution. The MaxEnt 1 software was used for mass deconvolution of the charge state envelopes.

Coiled coil association efficiency

The efficiency of coiled coil association of E-OVA323 to the 10% ELP/ELP-K was studied using E-OVA323-TMR. Coiled-coil micelles, plain ELP micelles mixed with E-OVA323-TMR and free E-OVA323-TMR samples were prepared containing 2 μM E-OVA323-TMR and 20 μM polypeptide. First, 175 μL of each sample was incubated at 37 °C for 5 minutes. Subsequently, unbound E-OVA323-TMR was removed using a centrifugal filter unit (Amicon Ultra-0.5 mL) at 10000 rcf and 37 °C for 5 minutes. The resulting solutions were washed five times with 175 μL 37 °C PB using the same centrifugation protocol. The samples were diluted with 300 μL PB and the fluorescence was measured using fluorescence spectroscopy.

Fluorescence spectroscopy

Fluorescence intensities were measured on an FS920 steady state spectrometer from Edinburgh Instruments using an excitation of 541 nm (slit size of 5 nm) and emission of 572 nm (slit size of 1 nm). A series of E-OVA323-TMR concentrations ranging from 62.5 nM to 2 μM was measured to confirm the linear correlation between fluorescent signal and TMR concentration.

LDH assay

The LDH assay was performed using the Lactate Dehydrogenase Activity Assay Kit (Sigma-Aldrich) per the manufacturer's instructions. The supernatants of BMDC cultures that had been incubated with the ELP-based samples to study uptake were tested to study the effect of each sample at the four highest concentrations tested (i.e. 10, 30, 90 and 270 nM OVA323) and compare the LDH activity to that in the supernatants of cells that had been incubated with media only.

LPS content determination

The endotoxin levels were determined using HEK-blue TLR4 reporter cells (InvivoGen) expressing either the human (ELP) or murine (ELP-K and ELP-OVA323) TLR4-MD2 receptor

complex. Suspensions of 25000 cells in 100 μL were mixed with 100 μL polypeptide or LPS samples at various concentrations in a 96 well plate (Greiner Bio-One). The plate was incubated at 37 °C overnight, resulting in the activation of the TLR4 pathway, which was detected by secretion of embryonic alkaline phosphatase (SEAP). The SEAP levels were measured with a QUANTI-Blue assay. First, 20 μL of the cell supernatants were incubated with 180 μL QUANTI-Blue solution (rep-qb1, InvivoGen) for 2 hours at 37 °C. Next, the optical density (OD) at 650 nm was measured with a Tecan i-control 1.7.1.12 plate reader. The endotoxin levels in the covalent, coiled-coil and hybrid micelles were <5 EU/mg.

Supplemental Figures and Tables

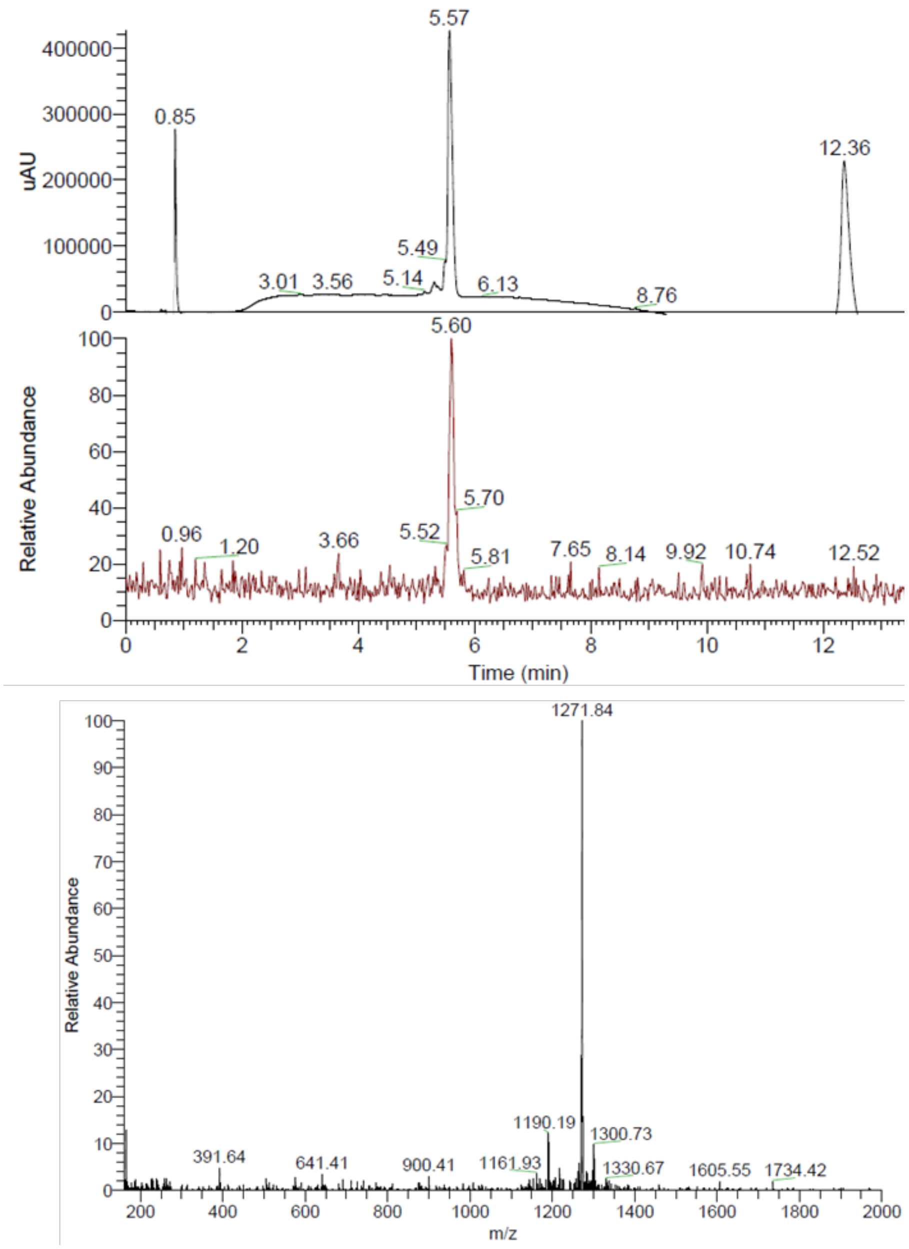

Figure S1. LC-MS spectrum of peptide E. Chromatogram is shown in the top and middle panels. Observed mass (bottom): 1271.84; calculated mass ($[M+2H^{+}]^{2+}$): 1272.97.

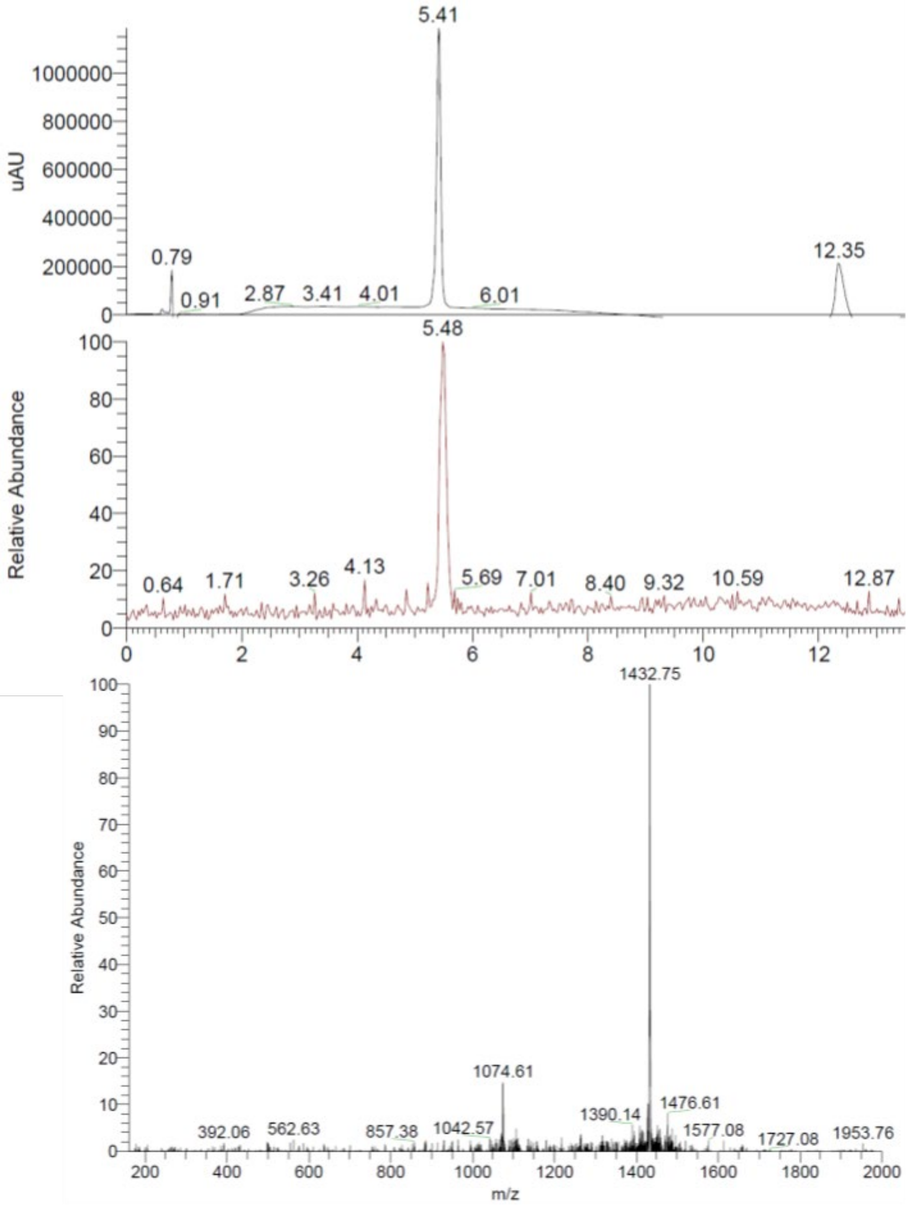

Figure S2. LC-MS spectrum of E-OVA323. Chromatogram is shown in the top and middle panels. Observed mass (bottom): 1432.89 and 1074.89; calculated mass ($[M+3H^{+}]^{3+}$ and $[M+4H^{+}]^{4+}$): 1434.28 and 1075.96.

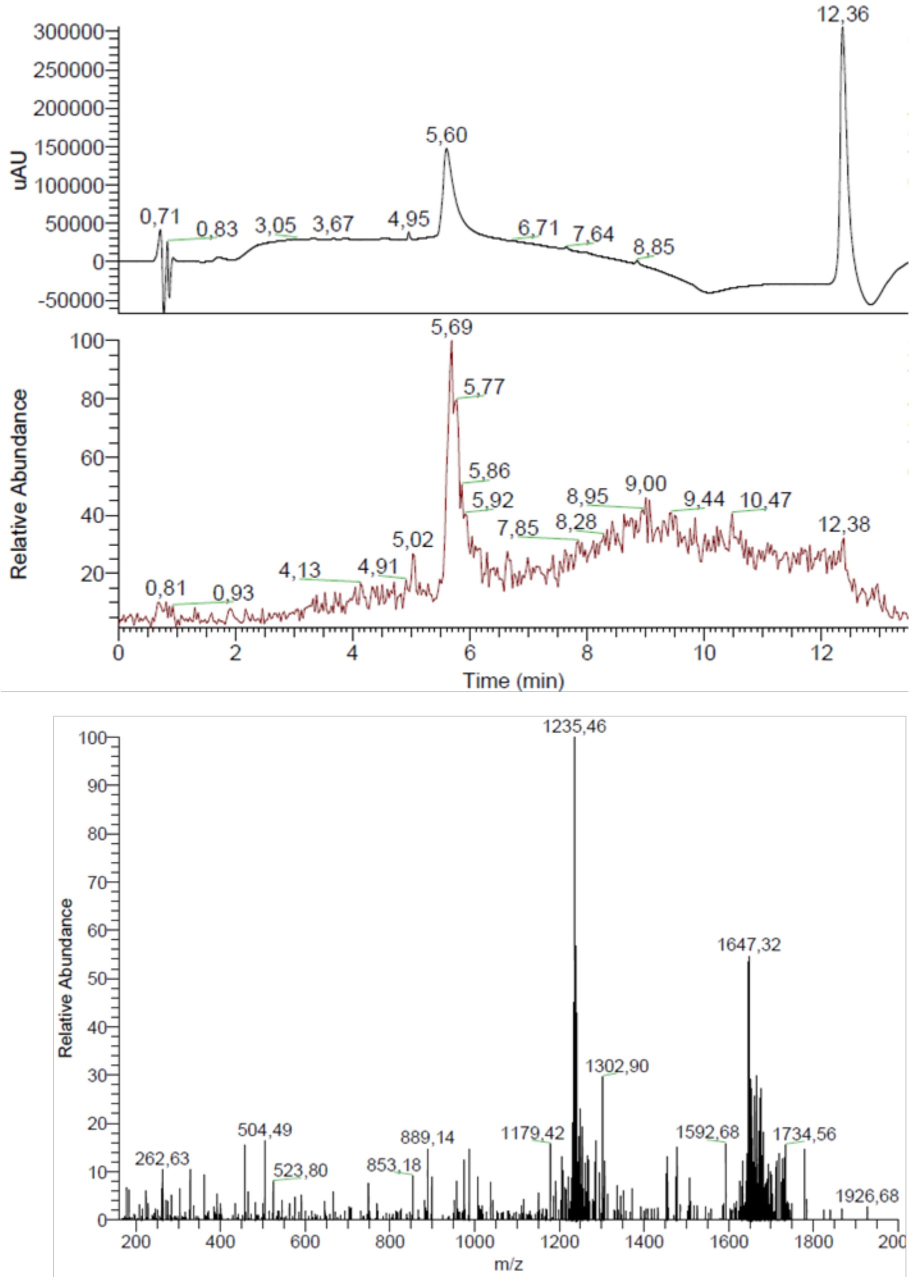

Figure S3. LC-MS spectrum of E-OVA323-TMR. Chromatogram is shown in the top and middle panels. Observed mass (bottom): 1647.32 and 1235.46; calculated mass ($[M+3H^+]^{3+}$ and $[M+4H^+]^{4+}$): 1648.18 and 1236.39.

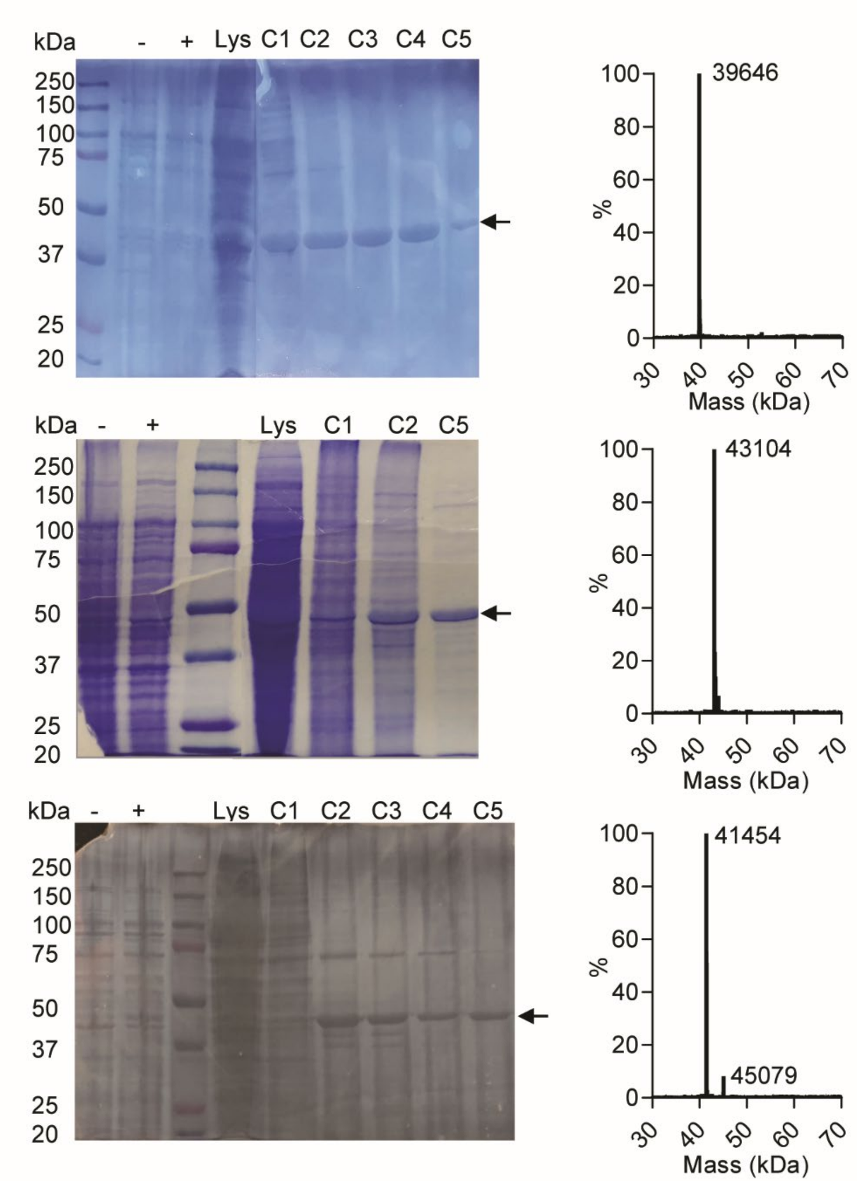

Figure S4. Expression, purification and identification of ELP (top) ELP-K (middle) and ELP-OVA323 (bottom). Left) Samples were analyzed on 10% SDS polyacrylamide gels with Coomassie Blue (ELP-K) or copper chloride (ELP and ELP-OVA323) staining. ELP, ELP-K and ELP-OVA323 are clearly visible at 40 kDa, 43 kDa and 40 kDa, respectively. In each gel, the black arrow marks the location of the purified polypeptide. Lanes marked with - and + are before and after adding IPTG; Lys = lysate; C1-5 refer to samples taken after 1-5 cycles of ITC. Right) Mass spectra matched the theoretical masses of ELP, ELP-K and ELP-OVA323 without the N-

terminal methionine. Expected masses were 39642 Da (ELP-E), 43100 Da (ELP-K) and 41456 Da (ELP-OVA323).

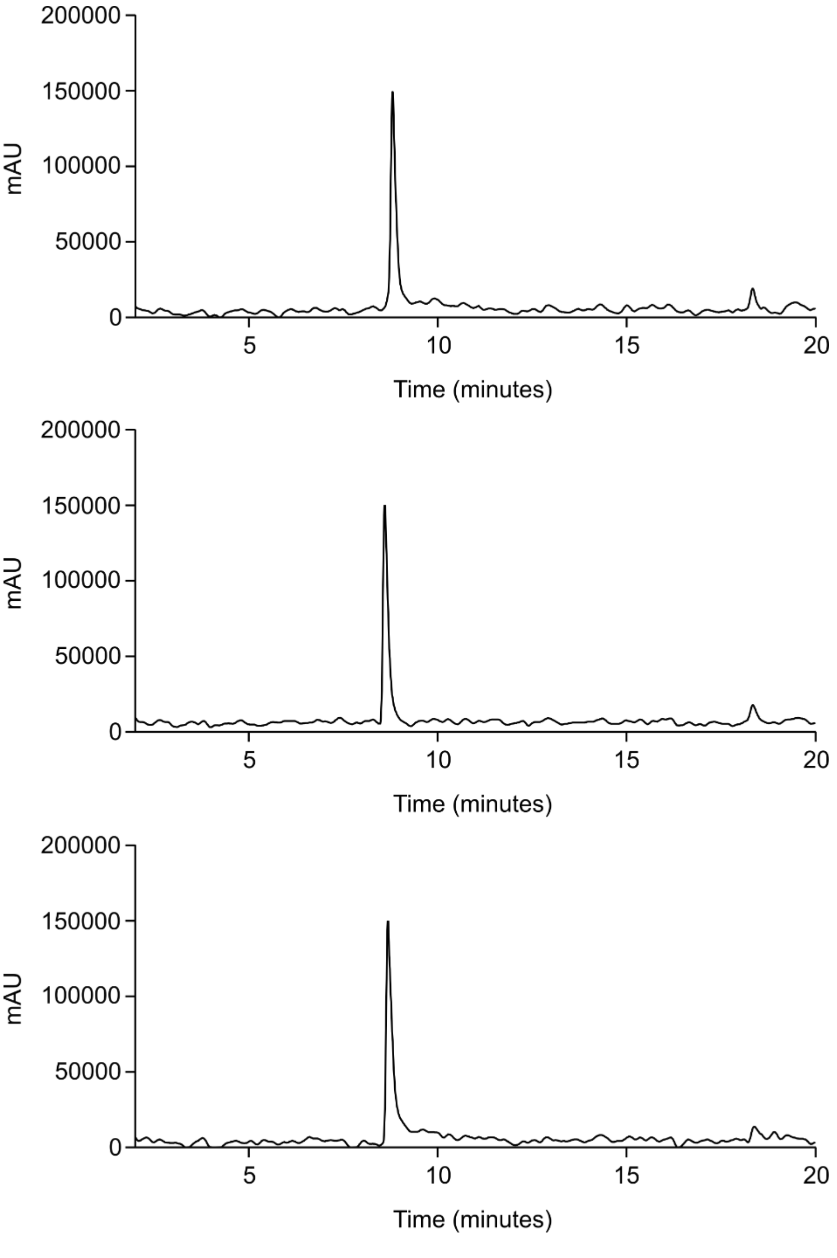

Figure S5. RP-HPLC analysis of ELP (top), ELP-K (middle) and ELP-OVA323 (bottom). Gradient of 0 to 90% acetonitrile in water containing 0.1% TFA. Traces were normalized to match the peak height of ELP.

Table S1. Composition and size of coiled coil-displaying ELP micelles

**Formulation**	**Without peptide E**		**With peptide E**	
	**D_h [nm]**	**PdI**	**D_h [nm]**	**PdI**
ELP	53.7 ± 0.7	0.033 ± 0.011	Not applicable	
5% ELP-K	55.3 ± 0.1	0.108 ± 0.021	55.1 ± 0.5	0.084 ± 0.030
10% ELP-K	60.8 ± 1.0	0.192 ± 0.017	55.0 ± 0.4	0.077 ± 0.013
20% ELP-K	129.9 ± 5.3	0.743 ± 0.177	56.6 ± 0.4	0.100 ± 0.006
30% ELP-K	618.3 ± 205.9	0.683 ± 0.083	58.4 ± 0.6	0.109 ± 0.027
40% ELP-K	Not measured		66.5 ± 2.9	0.186 ± 0.064
50% ELP-K	Not measured		143.3 ± 56.2	0.349 ± 0.075

[polypeptide] = 20 µM; T = 37 °C; measured in 10 mM PB, pH 7.8. All reported averages are based on three DLS measurements.

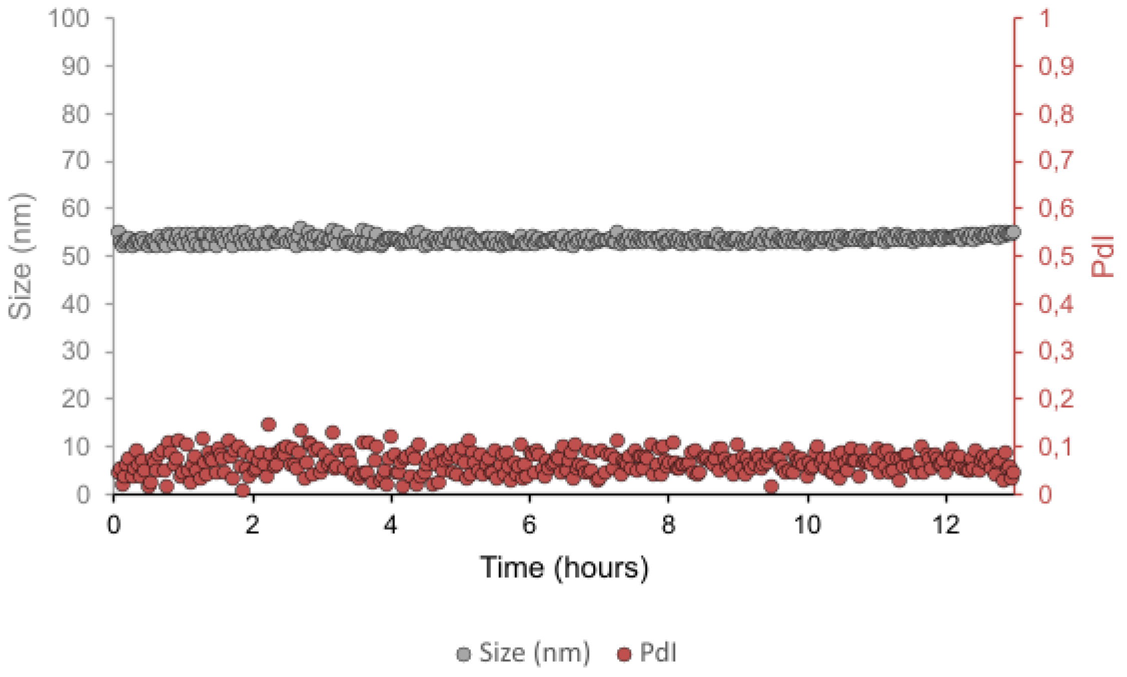

Figure S6. ELP micelles display stable hydrodynamic diameter over time. ELP micelles were incubated at 37 °C and DLS measurements were performed as a function of time. [ELP] = 10 µM in PBS (10 mM phosphate buffer, pH 7.8; 150 mM NaCl).

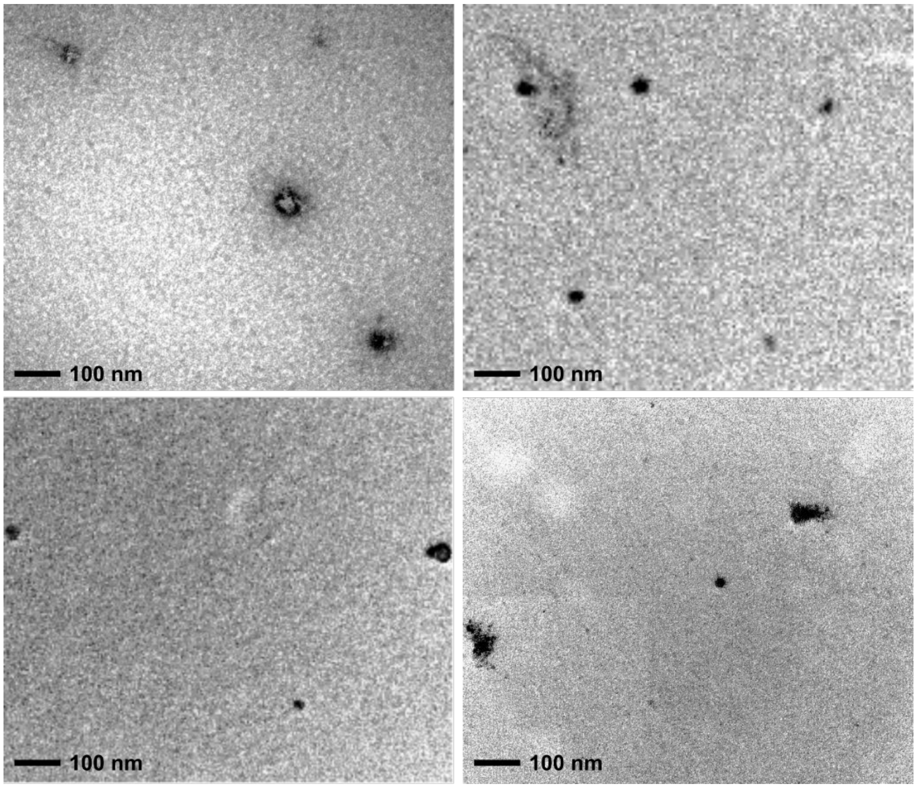

Figure S7. TEM imaging of ELP micelles displaying coiled coils. Left) ELP/ELP-E (9:1); right) ELP/ELP-K (9:1). Bottom) with an equimolar amount of the complementary peptide; top) without an equimolar amount of the complementary peptide. [polypeptide] = 20 μM in water; E/K ratio = 1; T = 37 °C; stained with 1% uranyl acetate.

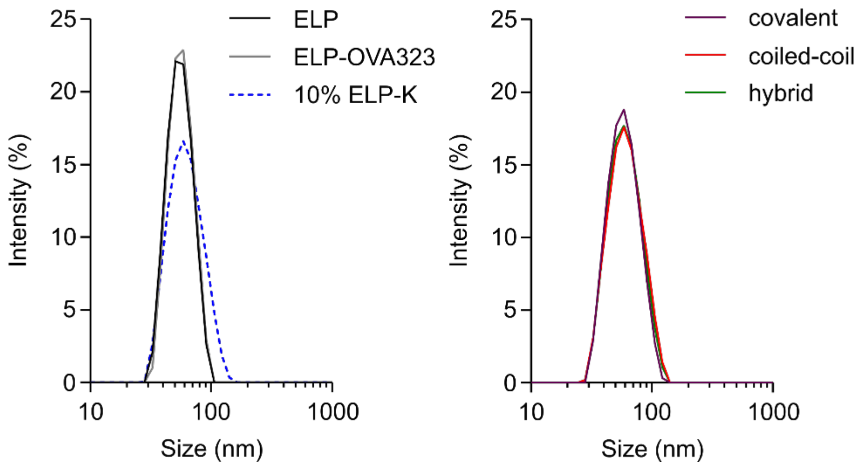

Figure S8. Hydrodynamic diameters of functionalized ELP micelles. [polypeptide] = 10 µM; T = 37 °C; measured in 10 mM PB, pH 7.8.

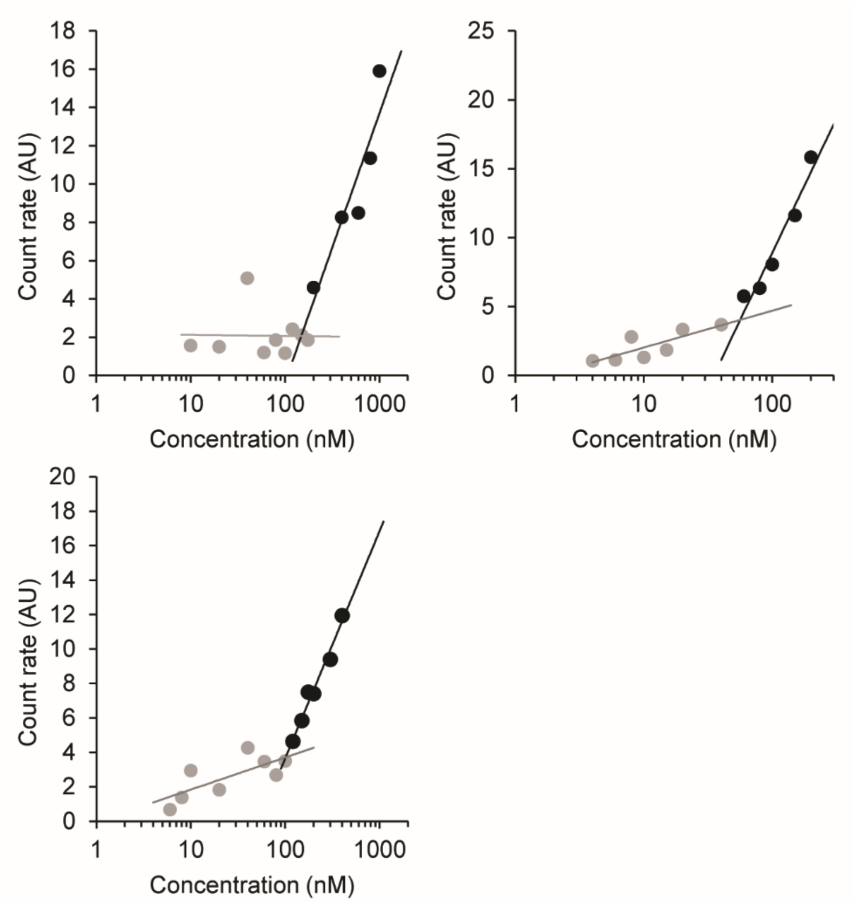

Figure S9. CMC determination of ELP (top left) and ELP-OVA323 (bottom left) and CAC determination of ELP-K (top right). Measured in 10 mM PB, pH 7.8, at 37 °C. Count rate was

measured by SLS as a function of [ELP], [ELP-K] or [ELP-OVA323]. Count rates were normalized to the count rate of the buffer. Gray data represent samples that did not contain particles according to the autocorrelation functions (see Figure S10); black data represent the samples for which the autocorrelation functions had a sigmoidal shape. The CMC of ELP was determined at 0.15 μM, the CAC of ELP-K was determined at 56 nM and the CMC of ELP-OVA323 was determined at 0.10 μM by calculating the concentration at the intercept of the trend lines.

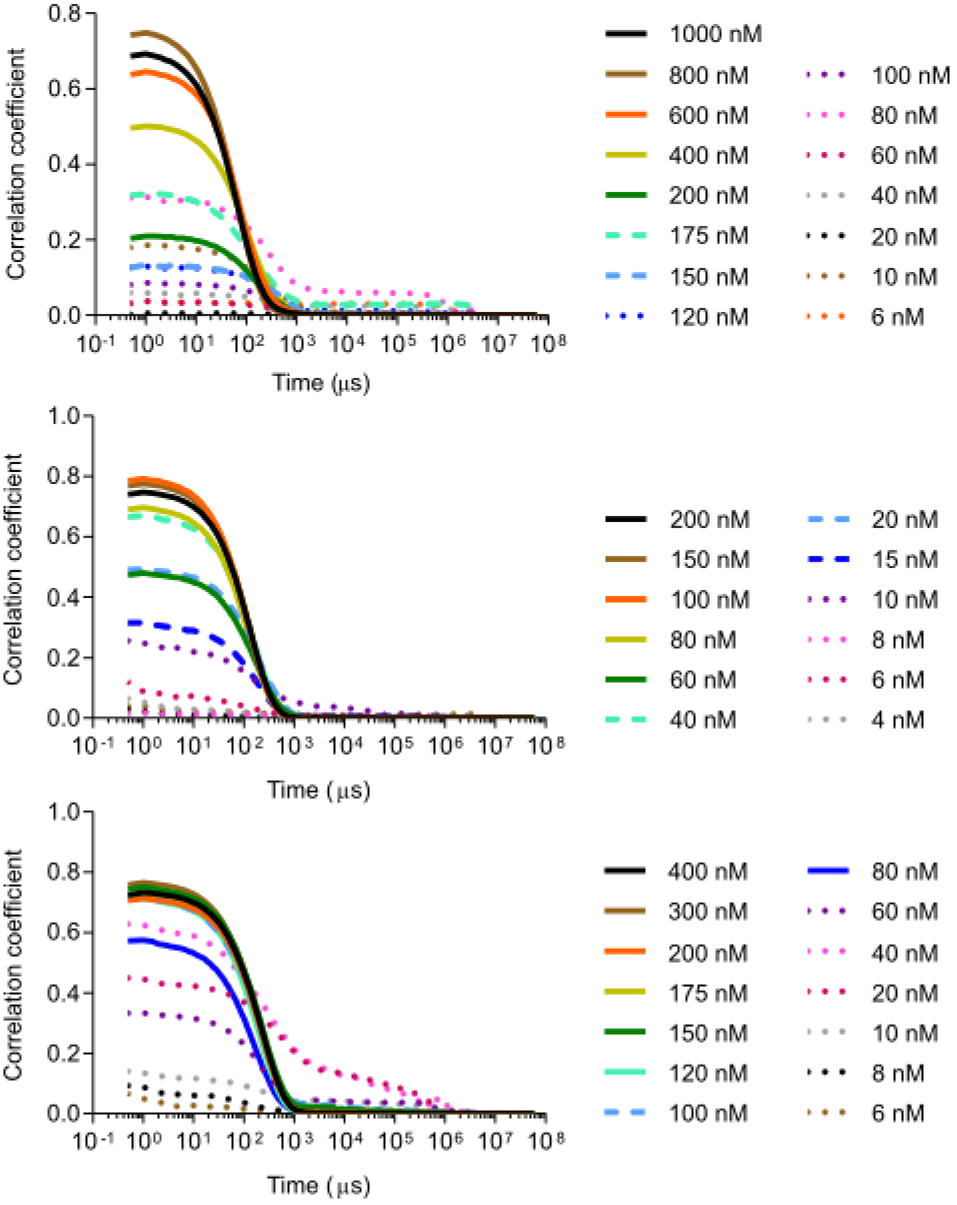

Figure S10. Autocorrelation functions of DLS measurements for CMC/CAC determinations of ELP (top), ELP-K (middle) and ELP-OVA323 (bottom). Each graph is an average of three measurements. Solid lines represent samples for which particles were detected; dotted lines represent samples for which particles could not be detected; dashed lines represent samples for which no particles were detected for the first measurement, but particles were detected for the last measurement.

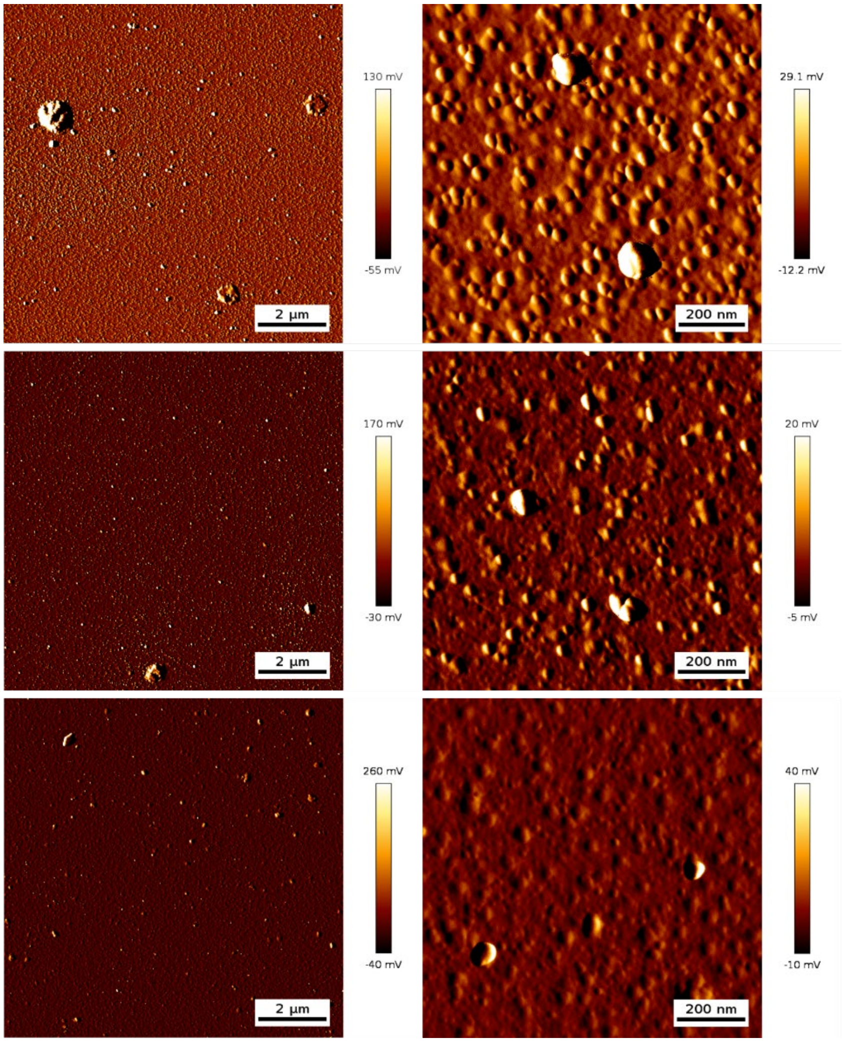

Figure S11. AFM images of covalent (top), coiled-coil (middle) and hybrid (bottom) micelles. [polypeptide] = 2 µM in water, deposited on a silicon oxide surface and dried at 37 °C; error trace mode; imaged using medium (left) and high (right) magnifications.

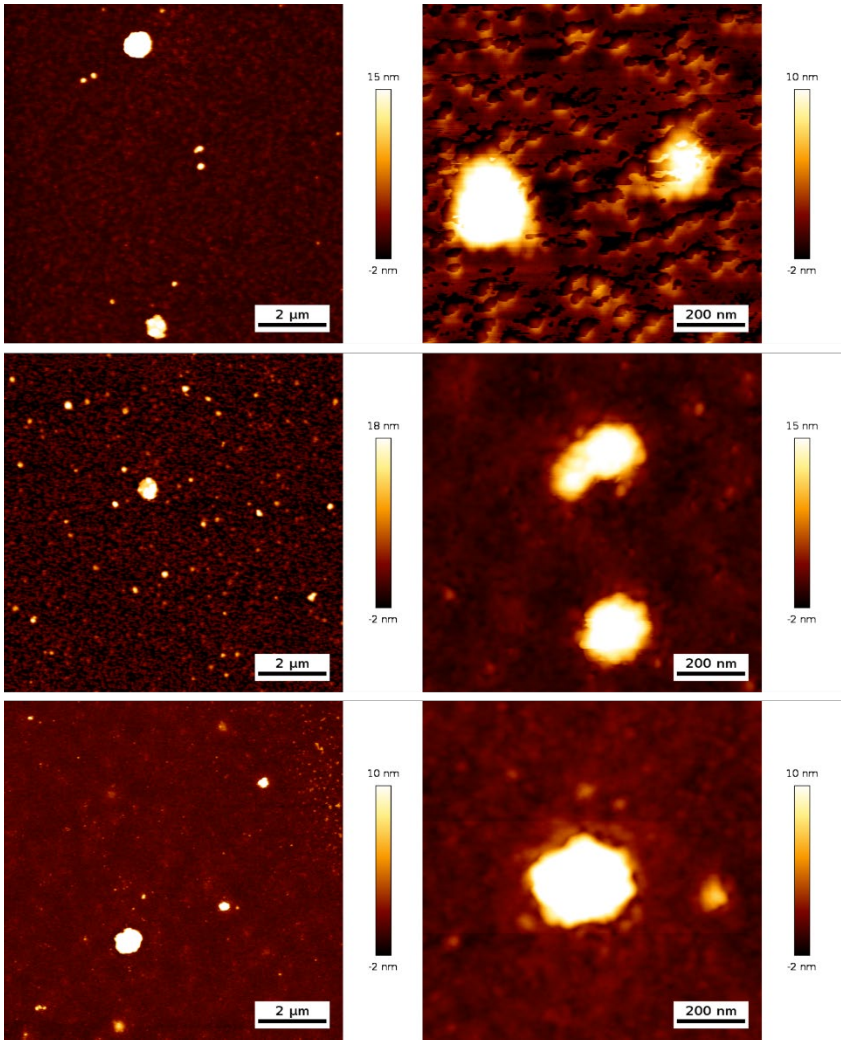

Figure S12. AFM images of covalent (top), coiled-coil (middle) and hybrid (bottom) micelles. [polypeptide] = 2 μM in water, deposited on a mica surface and dried at 37 °C; height trace mode; imaged using medium (left) and high (right) magnifications.

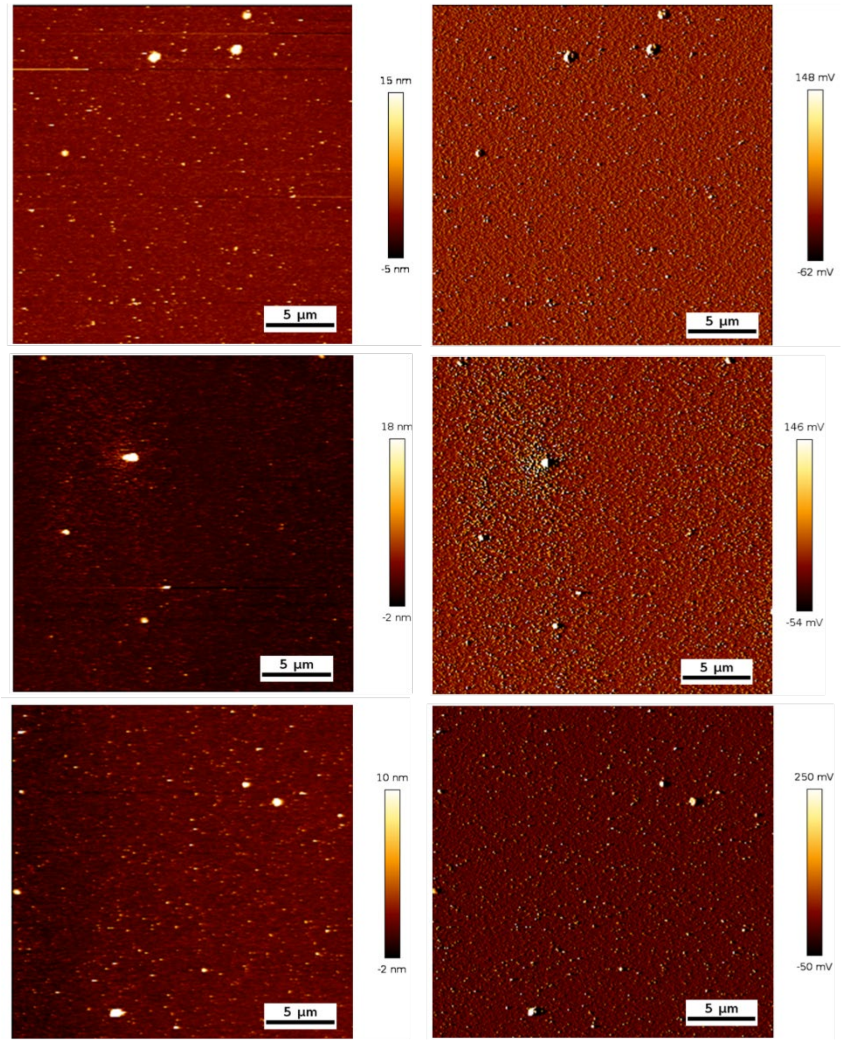

Figure S13. AFM images of covalent (top), coiled-coil (middle) and hybrid (bottom) micelles. [polypeptide] = 2 µM in water, deposited on a silicon oxide surface and dried at 37 °C; height trace (left) and error trace (right) mode; imaged using low magnifications.

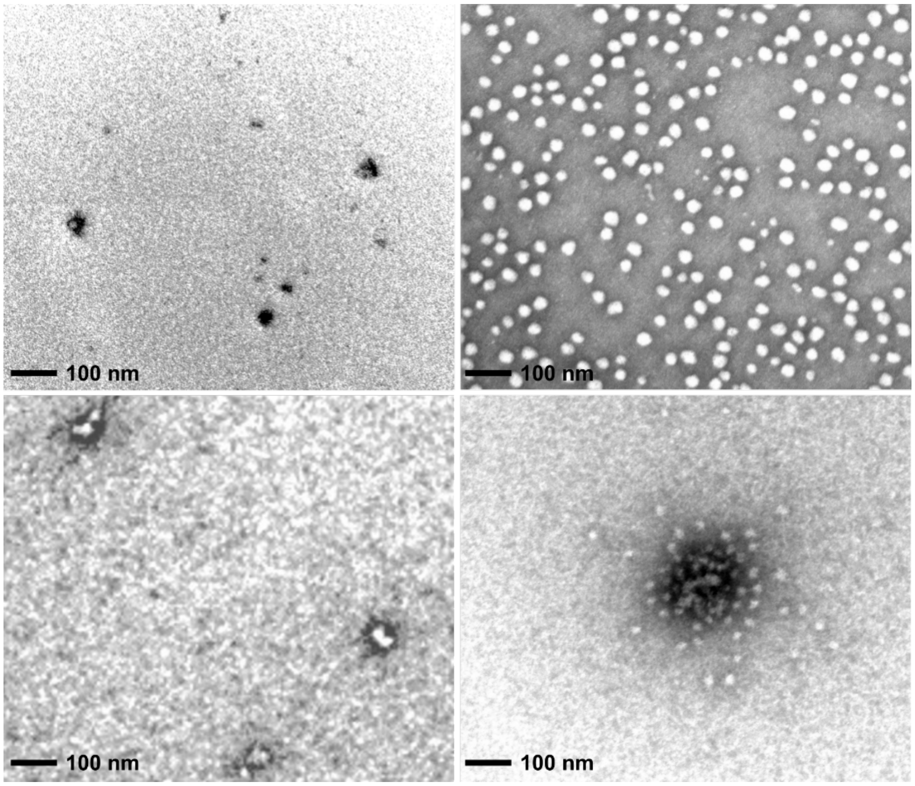

Figure S14. TEM imaging of covalent (top left), coiled-coil (top right) and hybrid (bottom left and right) micelles. [polypeptide] = 20 μM in water; E/K ratio = 1; T = 37 °C; stained with 1% uranyl acetate.

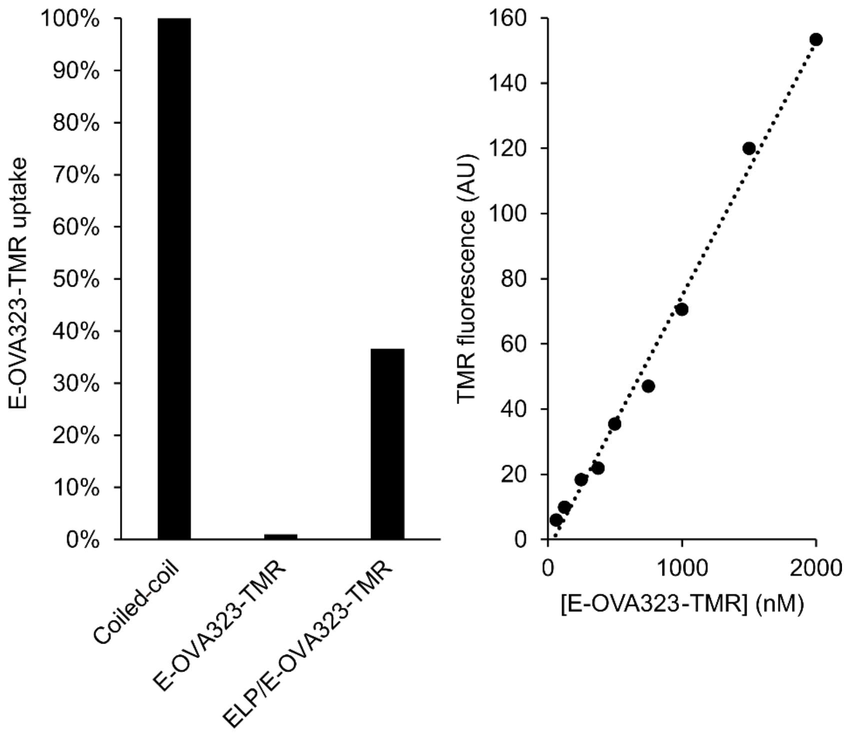

Figure S15. Coiled coil association efficiency on the ELP micelle surface. Left) [polypeptide] = 20 μM; [E-OVA-TMR] = 2 μM; separation of unbound E-OVA323 from micelle in the presence (coiled-coil) or absence (ELP/E-OVA323-TMR) of ELP-K was performed at 37 °C. The fluorescent signals of the resulting samples were normalized to the value of the coiled-coil sample.

Right) E-OVA323-TMR concentrations in the relevant range are linearly associated with the fluorescent signal.

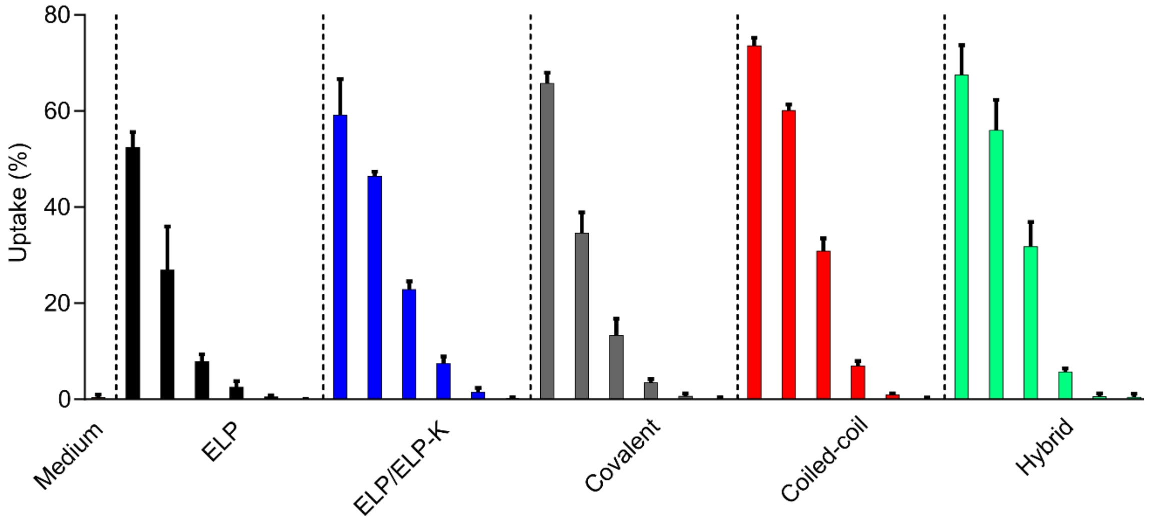

Figure S16. BMDC uptake of FITC-labeled formulations with and without ELP-K. BMDCs were incubated for 4 hours with fluorescently labeled micelles or as a negative control with media only. OVA323 concentrations were (from left to right) 270, 90, 30, 10, 3.3 and 1.1 nM, corresponding to 2700, 900, 300, 100, 33 and 11 nM polypeptide, respectively. The BMDCs were subsequently analyzed with flow cytometry. The gating strategy is shown in Figure S18. The bars depict the mean and standard deviation of fluorescently labeled cells.

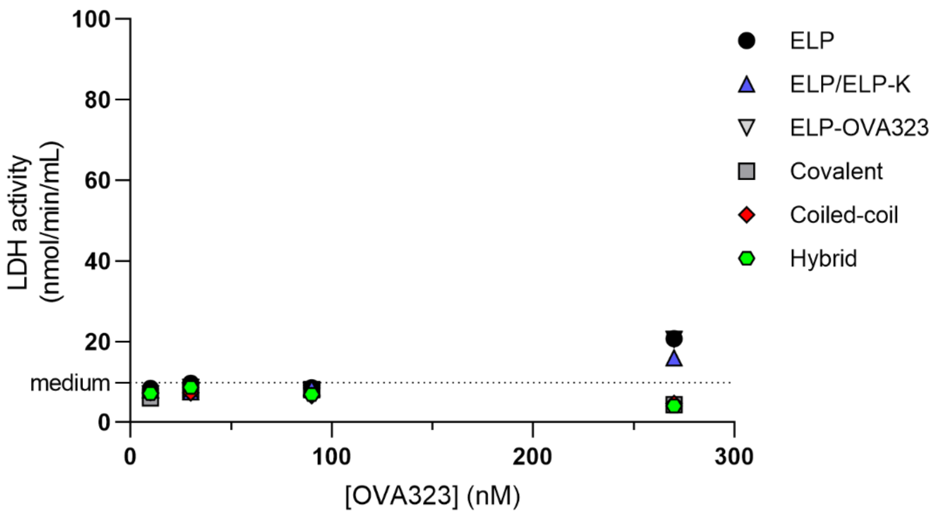

Figure S17. Lactate Dehydrogenase (LDH) activity remains concentration-independent in the supernatants of BMDCs incubated with hybrid, covalent, and coiled-coil formulations. The

supernatants were incubated with pyruvate and NAD, resulting in LDH-dependent formation of NADH, which is colorimetrically detected at 450 nm. The data points represent the mean LDH activity levels of 4 replicate samples. Only the highest concentration (i.e. 270 nM OVA323) of the ELP, ELP/ELP-K and ELP-OVA323 samples showed LDH activity slightly above the negative control level. The supernatants of these samples were tested on a separate plate from the supernatants of the Covalent, Coiled-coil, and Hybrid samples (containing the same components as the ELP, ELP/ELP-K and ELP-OVA323 samples), suggesting the difference in LDH activity level could be a plate-to-plate effect. The supernatants incubated with the highest sample concentrations were tested at a lowest dilution, which may have resulted in slightly higher background levels compared to the other supernatants.

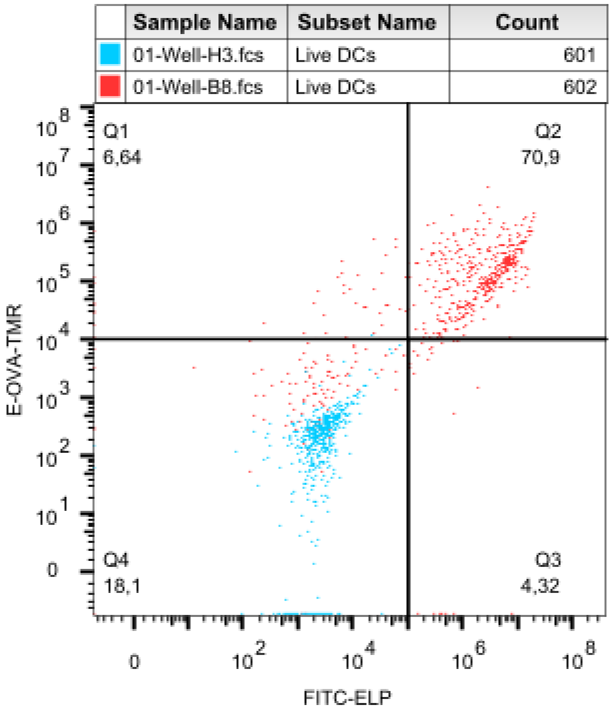

Figure S18. Representative TMR vs. FITC plot for the analysis of antigen and micelle uptake in DCs. Each DC is shown as a dot with the corresponding FITC and TMR signals. Gatings were chosen to divide the cells into four quadrants: FITC+, TMR+, double positive and double negative. A typical well of DCs pulsed with coiled-coil micelles is shown in red and the numbers show the percentages of cells in each quadrant. Blue dots represent a set of DCs pulsed with media only.

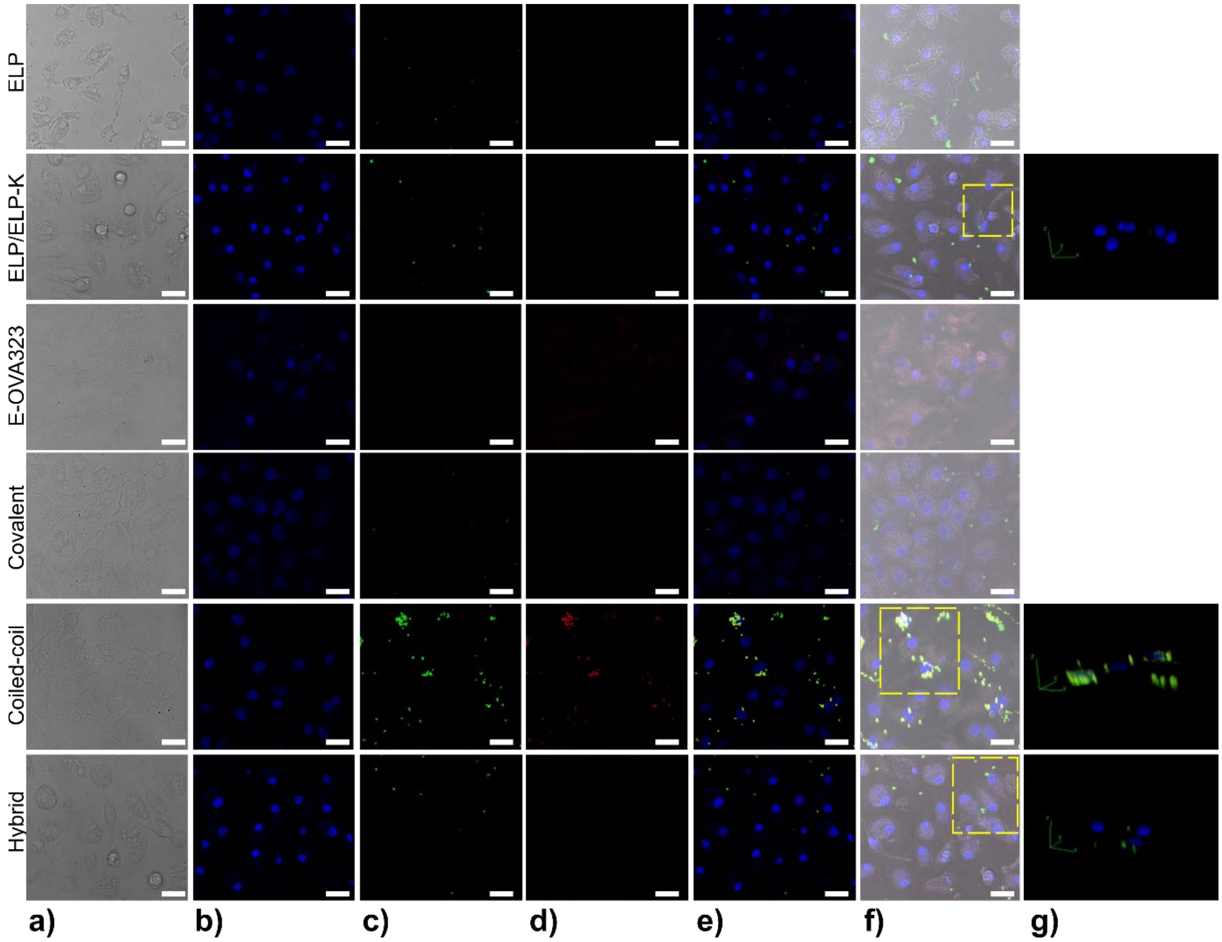

Figure S19. ELP-K-dependent uptake of ELP micelles into BMDCs. BMDCs were incubated for 4 hours with fluorescently-labeled peptide or micelles containing 90 nM OVA323 (900 nM ELP). The BMDCs were washed thoroughly with media to remove excess particles and imaged on a confocal microscope. Various images were taken from each sample: a) bright field image, b) blue channel (cell nuclei), c) green channel (ELP), d) red channel (E-OVA323). An overlay of the fluorescent channels is shown in (e). An overlay of the Z-stacks of the bright field and fluorescent channels is shown in (f). A three-dimensional image of the fluorescent channels is shown in (g) for the cultures in which uptake was observed (taken from the area indicated with yellow squares in (f)). Scale bar = 25 μM.

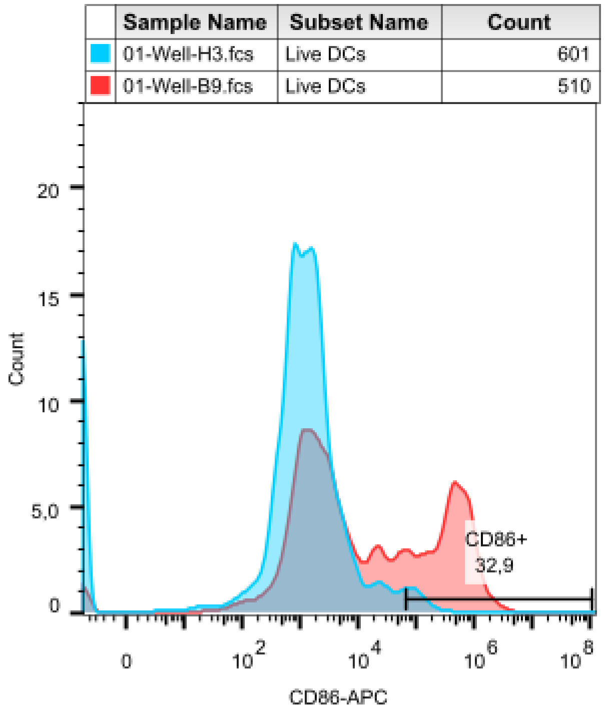

Figure S20. Representative CD86 histogram plot. DCs are plotted in a histogram corresponding to their CD86-APC signal. The gating for CD86+ cells is shown as a black bar. A typical well of

DCs pulsed with coiled-coil micelles is shown in red together with the percentage of CD86+ DCs (32.9%). The blue histogram represents a set of DCs pulsed with media only.

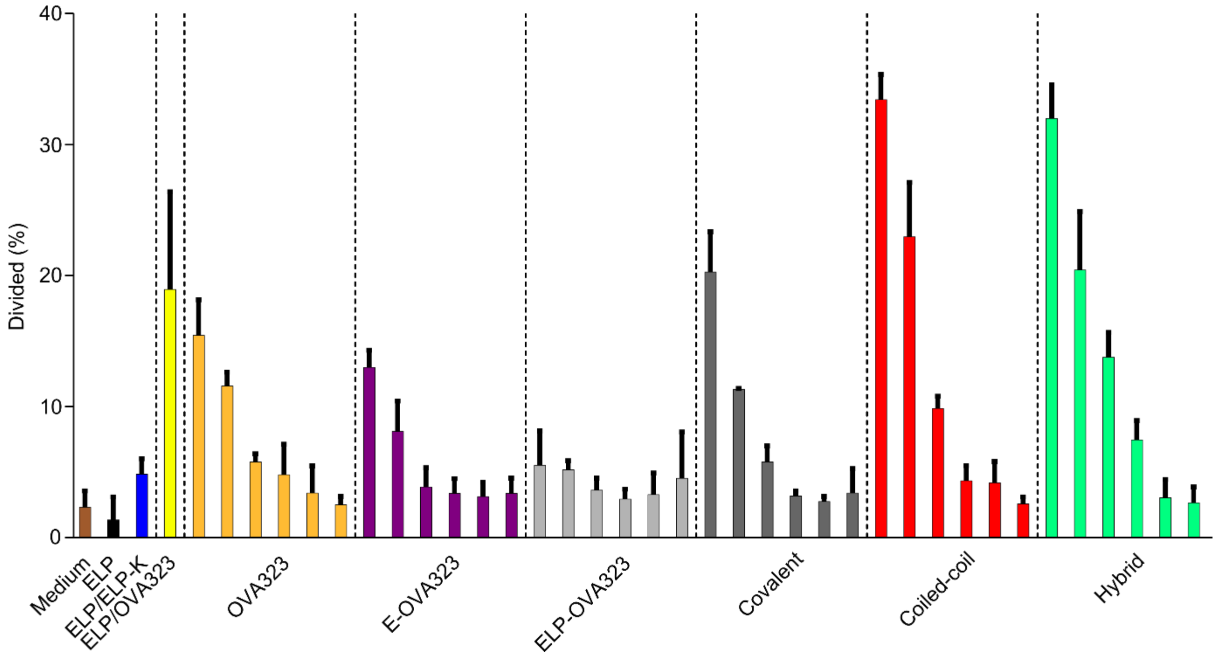

Figure S21. Proliferation of OT-II cells *in vitro*. BMDCs were incubated for 4 hours with micelles, peptide or with media only. OVA323 concentrations were (from left to right) 270, 90, 30, 10, 3.3 and 1.1 nM, corresponding to 2700, 900, 300, 10, 33 and 11 nM polypeptide, respectively. The BMDCs were subsequently exposed for 3 days to OT-II cells containing fluorescent dye. The decrease of fluorescent signal in the OT-II population was then analyzed with flow cytometry. The gating strategy is depicted in Figure S22. As negative controls, BMDCs were pulsed with media, plain ELP micelles or ELP/ELP-K micelles (containing 10% ELP-K). ELP micelles mixed with free OVA323 peptide ("ELP/OVA323") was included as a positive control. The polypeptide concentrations of ELP and ELP-K and ELP/OVA323 was 2.7 μM, matching the highest polypeptide concentration of the covalent, coiled-coil and hybrid groups. The bars depict the mean and standard deviation of the divided cells.

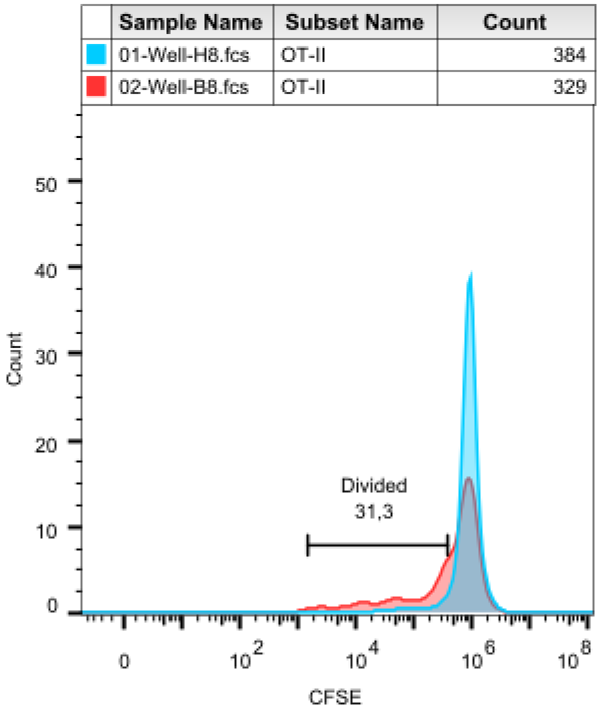

Figure S22. Representative CSFE dilution plot. CD4^{+} T-cells are plotted in a histogram corresponding to their CFSE signal. The gating for cells with diluted CFSE (divided cells) is shown as a black bar. A typical well of T-cells cocultured with coiled-coil micelle pulsed DCs is shown in red together with the percentage of divided cells (31.3%). The blue histogram represents T-cells cocultured with media pulsed DCs.
